# Cell-free DNA reveals distinct pathology of multisystem inflammatory syndrome in children

**DOI:** 10.1172/JCI171729

**Published:** 2023-11-01

**Authors:** Temesgen E. Andargie, Katerina Roznik, Neelam Redekar, Tom Hill, Weiqiang Zhou, Zainab Apalara, Hyesik Kong, Oren Gordon, Rohan Meda, Woojin Park, Trevor S. Johnston, Yi Wang, Sheila Brady, Hongkai Ji, Jack A. Yanovski, Moon K. Jang, Clarence M. Lee, Andrew H. Karaba, Andrea L. Cox, Sean Agbor-Enoh

**Affiliations:** 1 Genomic Research Alliance for Transplantation (GRAfT) and Laboratory of Applied Precision Omics, National Heart, Lung, and Blood Institute (NHLBI), NIH, Bethesda, Maryland, USA. GFAfT is detailed in Supplemental Acknowledgments.; 2 Department of Biology, Howard University, Washington DC, USA.; 3 Department of Medicine, Johns Hopkins University, School of Medicine, Baltimore, Maryland, USA.; 4 Integrated Data Sciences Section, Research Technologies Branch, National Institute of Allergy and Infectious Diseases (NIAID), NIH, Bethesda, Maryland, USA.; 5 Department of Biostatistics, Bloomberg School of Public Health, Johns Hopkins University, Baltimore, Maryland, USA.; 6 Infectious Diseases Unit, Department of Pediatrics, Hadassah Medical Center, Faculty of Medicine, Hebrew University of Jerusalem, Jerusalem, Israel.; 7 Section on Growth and Obesity, Eunice Kennedy Shriver National Institute of Child Health and Human Development (NICHD), NIH, Bethesda, Maryland, USA.

**Keywords:** Infectious disease, Inflammation, Cytokines, Epigenetics, Molecular diagnosis

## Abstract

Multisystem inflammatory syndrome in children (MIS-C) is a rare but life-threatening hyperinflammatory condition induced by infection with severe acute respiratory syndrome coronavirus 2 (SARS-CoV-2) that causes pediatric COVID-19 (pCOVID-19). The relationship of the systemic tissue injury to the pathophysiology of MIS-C is poorly defined. We leveraged the high sensitivity of epigenomics analyses of plasma cell-free DNA (cfDNA) and plasma cytokine measurements to identify the spectrum of tissue injury and glean mechanistic insights. Compared with pediatric healthy controls (pHCs) and patients with pCOVID-19, patients with MIS-C had higher levels of cfDNA primarily derived from innate immune cells, megakaryocyte-erythroid precursor cells, and nonhematopoietic tissues such as hepatocytes, cardiac myocytes, and kidney cells. Nonhematopoietic tissue cfDNA levels demonstrated significant interindividual variability, consistent with the heterogenous clinical presentation of MIS-C. In contrast, adaptive immune cell–derived cfDNA levels were comparable in MIS-C and pCOVID-19 patients. Indeed, the cfDNA of innate immune cells in patients with MIS-C correlated with the levels of innate immune inflammatory cytokines and nonhematopoietic tissue–derived cfDNA, suggesting a primarily innate immunity–mediated response to account for the multisystem pathology. These data provide insight into the pathogenesis of MIS-C and support the value of cfDNA as a sensitive biomarker to map tissue injury in MIS-C and likely other multiorgan inflammatory conditions.

## Graphical abstract

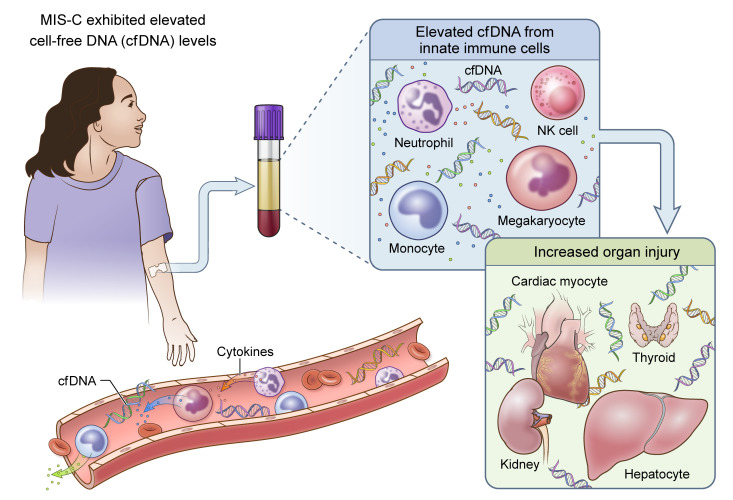

## Introduction

Multisystem inflammatory syndrome in children (MIS-C) is a rare yet life-threatening hyperinflammatory condition induced by infection with severe acute respiratory syndrome coronavirus 2 (SARS-CoV-2) (1). MIS-C shares clinical features with those of pediatric patients with severe COVID-19 (pCOVID-19) (2), including multiorgan pathology. While MIS-C and pCOVID-19 are not difficult to distinguish clinically, the heterogeneous, multiorgan tissue injury observed in patients with MIS-C resembles other childhood inflammatory disorders for which the mechanistic understanding, diagnosis, and treatment are less clear. Given the severity and heterogeneity of MIS-C symptomatology, there is a pressing need for early monitoring of systemic injury and inflammation in these patients to gain an understanding of the drivers of MIS-C immunopathology.

Circulating molecules of cell-free DNA (cfDNA) are short fragments from nuclear or mitochondrial genomes released from dying or injured cells. cfDNA is present at low concentrations in the blood of healthy individuals, and levels are significantly elevated during pathological conditions (3–6). Owing to a short half-life in the blood (15–20 minutes) (7), cfDNA can serve as a real-time marker of a dynamic disease process. In adult COVID-19, increased nuclear cfDNA (n-cfDNA) and mitochondrial cfDNA (mt-cfDNA) correlated with disease severity and adverse outcomes (8–10), highlighting the value of cfDNA as an early and sensitive predictor of disease progression. Collection of both mt-cfDNA and n-cfDNA allows one to account for the different patterns observed (9, 10). Unlike n-cfDNA, mt-cfDNA can be released from intact physiological cells, not necessarily from injured or dying cells (11). The n-cfDNA released into the circulation is bound to nucleosomes and is protected from deoxyribonuclease degradation. mt-cfDNA is poorly protected and prone to degradation in the plasma (11). Plasma cfDNA carries stable DNA methylation signatures related to its tissues of origin (12–14). The tissue-specific DNA methylation signatures can be surveyed to identify the cfDNA tissue source. In COVID-19, plasma tissue–specific cfDNA, measured via whole-genome bisulfite sequencing, correlates with clinical markers of end-organ injury, disease severity, and outcomes (9).

The pathogenesis and triggers of tissue injury in MIS-C are not fully understood. Existing studies indicate that circulating inflammatory cells and cytokines contribute to the progression of MIS-C (15–19). Conversely, other studies reported normal levels of circulating innate immune cells (20) and antibody responses in MIS-C, (21) highlighting the need to understand immune cell dynamics using alternative markers. Integrated analysis of cfDNA levels and immune processes (e.g., cytokine production) may provide a reliable marker to map the sources and severity of tissue injury in MIS-C and pCOVID-19. Of note, as a danger-associated molecular pattern, cfDNA activates innate immune cells and triggers a proinflammatory response (22–27). cfDNA is a major structural component of neutrophil extracellular traps (NETs) (28) that are expelled by activated neutrophils and trigger a coagulation cascade resulting in greater disease severity (29–31).

In this study, we performed whole-genome bisulfite sequencing to measure cell- or tissue-specific cfDNA as a measure of tissue injury in MIS-C and pCOVID-19 patients. Early identification of multiorgan tissue injury and a heightened innate immune response may lead to mechanistic insights to guide the diagnosis and treatment of MIS-C, pCOVID-19, and potentially other inflammatory diseases of childhood.

## Results

### Patient characteristics and clinical features.

**Figure 1:**
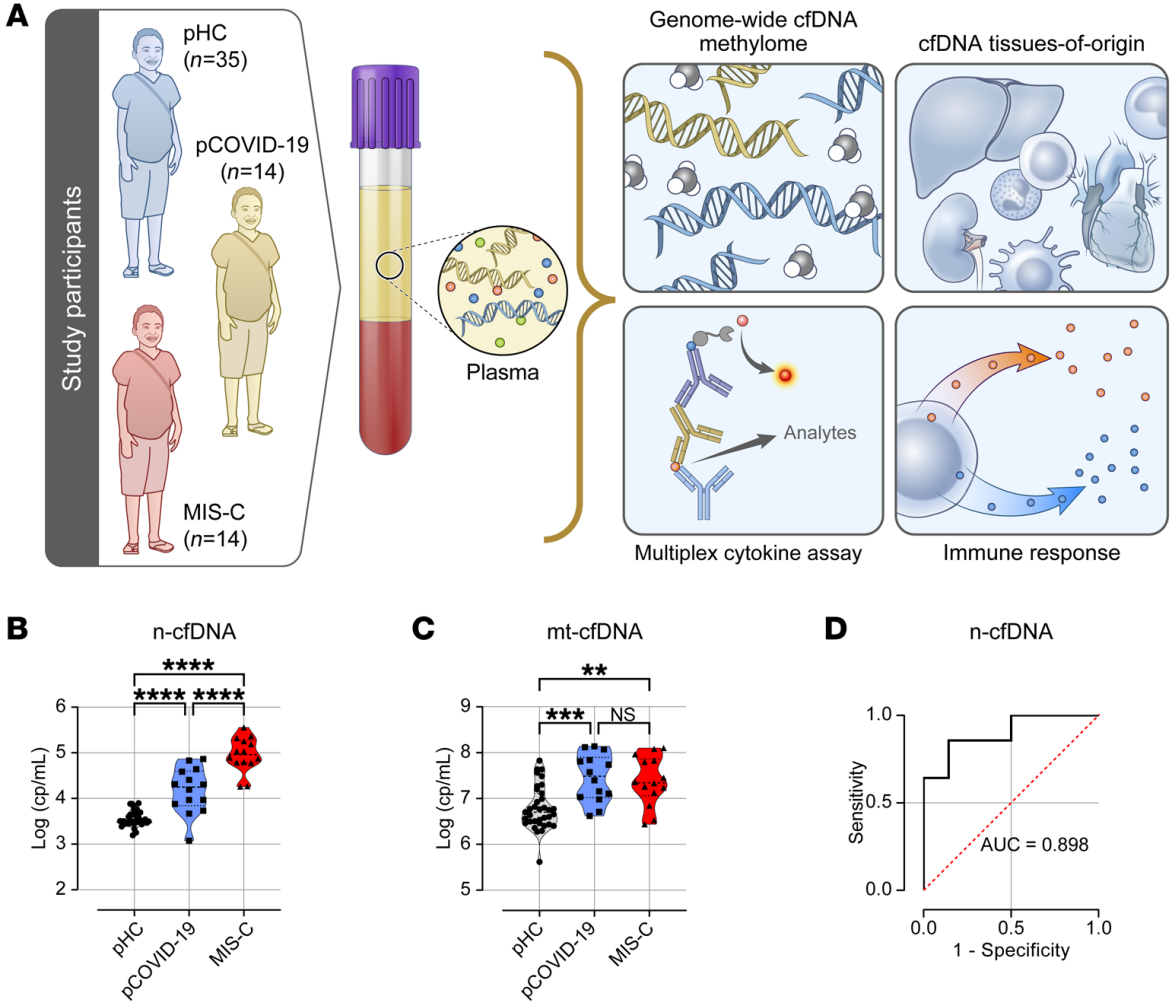
Elevated total cfDNA in patients with MIS-C. (**A**) Overview of study design and experimental workflow. (**B** and **C**) Concentrations of plasma n-cfDNA and mt-cfDNA in pHCs, patients with pCOVID-19, and patients with MIS-C. (**D**) ROC curve of n-cfDNA levels to distinguish MIS-C and pCOVID-19. cfDNA values are presented as cp/mL of plasma (log_10_-transformed). *P < 0.05, **P < 0.01, ***P < 0.001, and ****P < 0.0001, by Kruskal-Wallis test followed by Dunn’s multiple comparisons to compare tissue-specific cfDNA profiles among groups (**B** and **C**).

To define tissue injury patterns and inflammatory responses in patients with MIS-C and pCOVID-19, we performed an integrated analysis of plasma cfDNA and cytokines (Figure 1A). Our analysis included 28 pediatric patients (14 with acute pCOVID-19 and 14 with MIS-C) and 35 pediatric healthy controls (pHCs). We collected baseline plasma samples from patients with pCOVID-19 and MIS-C upon admission to the Johns Hopkins Hospital; pHC data were collected at outpatient clinic visits. Baseline demographic and clinical characteristics, laboratory results, treatments, and outcomes are shown in Table 1. No significant differences were observed in age, sex, race or ethnicity, or BMI among the MIS-C, pCOVID-19, and pHC groups. However, we observed significant baseline (upon hospital admission) elevations in the coagulation marker D-dimer (*P* = 0.026) and the cardiac marker pro-BNP (*P* = 0.032) in patients with MIS-C patients compared with patients with pCOVID-19. Peripheral white blood counts and other inflammatory markers were not different between the 2 groups. During hospitalization (days after admission), patients with MIS-C had significantly higher peak levels of D-dimer, C-reactive protein (CRP), troponin I (TnI), and pro–B-type natriuretic peptide (proBNP), as well as lower nadirs of lymphocytes and platelets, compared with patients with pCOVID-19 (*P* < 0.05 for all). Patients with MIS-C were substantially more likely to receive intravenous immune globulin (IVIG) treatment compared with patients with pCOVID-19 (85.7% and 7.1%, respectively).

### Levels of cfDNA are higher in patients with MIS-C compared with patients with pCOVID-19.

We hypothesized that MIS-C and pCOVID-19 show different tissue injury profiles that are indicative of distinct pathogenic mechanisms. We leveraged cfDNA as a sensitive biomarker to quantify the burden and sources of tissue injury in MIS-C and pCOVID-19. We first isolated cfDNA (Figure 1A) and confirmed its cfDNA quality, which revealed an expected nucleosomal distribution with a predominant peak at 167 bp corresponding to mononucleosome-bound DNA fragments (Supplemental Figure 1A; supplemental material available online with this article; https://doi.org/10.1172/JCI171729DS1). We then quantified total plasma n-cfDNA and mt-cfDNA, measures of global cellular damage or death, by digital-droplet PCR using primers and probes targeting the nuclear and mitochondrial genomes, respectively. Strikingly, n-cfDNA concentrations were 5.2 and 30 times higher, respectively, in patients with MIS-C (median [IQR]: 92,667 [60,819–187,788] cp/mL) compared with patients with pCOVID-19 (median [IQR]: 17,882 [7,036–39,934] cp/mL) and pHCs (median [IQR]: 3,072 [2,888–4,743] cp/mL) (Figure 1B; *P* < 0.001). Notably, the median n-cfDNA level (Figure 1B; *P* < 0.001) in patients with pCOVID-19 was also higher than that in pHCs. Within the MIS-C group, patients with clinical indices suggestive of severe disease demonstrated higher n-cfDNA compared with patients without the indices (Supplemental Table 1). Levels of mt-cfDNA were also significantly higher in both MIS-C and pCOVID-19 patients as compared with pHCs (Figure 1C; *P* < 0.01 for both), but there was no significant difference between the MIS-C and pCOVID-19 groups.

We next evaluated the ability of cfDNA levels to distinguish children with MIS-C from those with pCOVID-19. A receiver operator characteristic (ROC) curve analysis revealed good performance with an AUC of 0.89 (95% CI = 0.86–0.94) (Figure 1D; *P* = 0.0003) to discriminate patients with MIS-C from those with pCOVID-19. Overall, patients with MIS-C had elevated total plasma cfDNA levels that, alone, indicated greater tissue injury compared with patients with pCOVID-19 and pHCs.

### MIS-C has a distinct innate immune cell cfDNA pattern compared with pCOVID-19.

**Figure 2:**
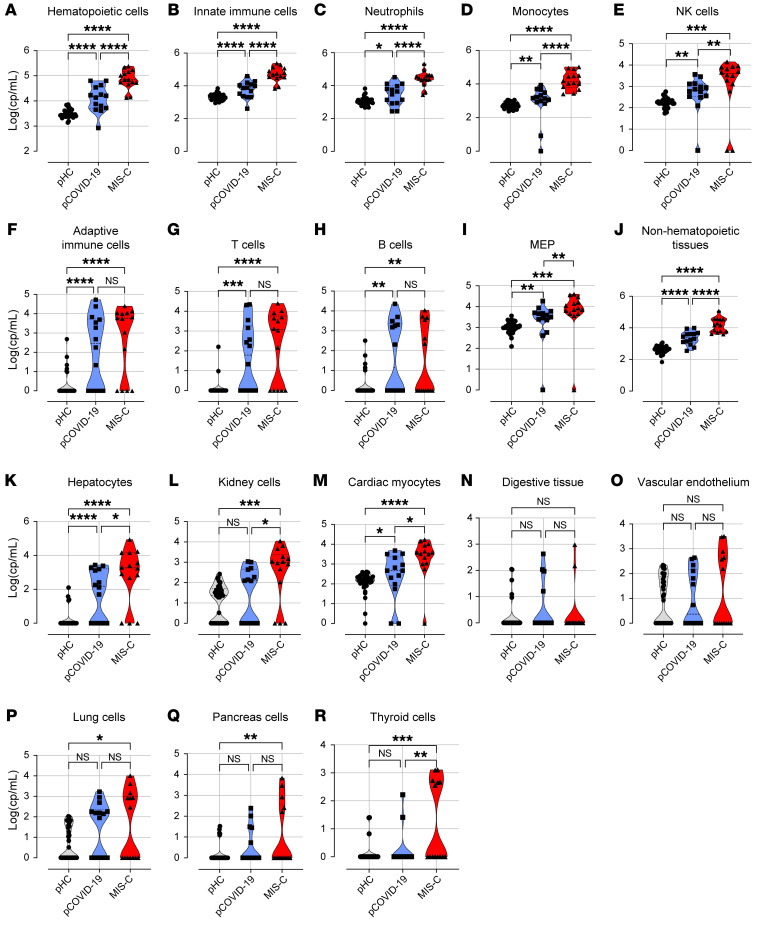
Exaggerated tissue injury pattern in patients with MIS-C. (**A**–**R**) Comparison of tissue-specific cfDNA derived from hematopoietic cells (**A**), innate immune cells (**B**), neutrophils (**C**), monocytes (**D**), NK cells (**E**), adaptive immune cells (**F**), T cells (**G**), B cells (**H**), MEPs (**I**), nonhematopoietic tissues (**J**), hepatocytes (**K**), kidney cells (**L**), cardiac myocytes (**M**), digestive tissue (**N**), vascular endothelium (**O**), lung (**P**), pancreas cells (**Q**), and thyroid cells (**R**) in pHCs (*n* = 35), patients with pCOVID-19 (*n* = 14), and patients with MIS-C (*n* = 14). Tissue-specific cfDNA values are presented as cp/mL or copies/mL of plasma (log_10_-transformed). *P < 0.05, **P < 0.01, ***P < 0.001, and ****P < 0.0001, by Kruskal-Wallis test followed by Dunn’s multiple comparisons to compare tissue-specific cfDNA profiles among groups.

We next sought to identify tissue sources for increased cfDNA levels observed in MIS-C. After isolation and bisulfite treatment, cfDNA maintained the expected nucleosomal distribution (Supplemental Figure 1B). We performed whole-genome bisulfite sequencing to an average 169 ± 3.1 million reads per sample, resulting in a uniquely averaged mapping efficiency of 89.53%. We observed high bisulfite conversion efficiency (99.95% ± 0.004%). We leveraged a library of tissue-/cell-specific methylation signatures of the 25 major cell or tissue types commonly involved in various disease conditions. Using this library, we performed deconvolution analysis using a meth_atlas algorithm (12) to deduce the relative contributions of different cell and tissue types to the plasma cfDNA pool. The fraction of each cell/tissue type was multiplied by the total n-cfDNA concentration to compute the absolute copy number of tissue-/cell-specific cfDNA. In all patient groups, hematopoietic cells were the major producers of cfDNA (Supplemental Figure 2A). Importantly, cfDNA derived from hematopoietic cells was increased in MIS-C compared with pCOVID-19 or pHCs (Figure 2A; *P* < 0.001).

Given the conflicting evidence about the frequency of circulating immune cells in MIS-C (15–18, 20), we compared complete blood count results and cfDNA from different immune cell types. The cfDNA profile exposed differences between MIS-C and pCOVID-19 that were not captured from the complete blood count. Absolute neutrophil count and platelet counts in the complete blood count were similar between MIS-C and pCOVID-19 (Supplemental Figure 2B). Strikingly, cfDNA levels from innate immune cells were significantly higher in patients with MIS-C than in patients with pCOVID-19 or pHCs (Figure 2B; *P* < 0.001). In particular, cfDNA levels from neutrophils, monocytes, and NK cells were significantly elevated in neutrophils, monocytes, and NK cells from patients with MIS-C (Figure 2, C–E). To gain further insight into the potential contribution of the adaptive immune system, we analyzed cfDNA levels derived from T cells and B cells. In both MIS-C and pCOVID-19 patients (compared with pHCs), B cell– and T cell–derived cfDNA levels were significantly elevated, either combined (Figure 2F; *P* < 0.001) or separately (Figure 2, G and H; *P* < 0.01 for both), However, the levels of B cell– and T cell–derived cfDNA were not different between MIS-C and pCOVID-19 patients (Figure 2, F–H), suggesting that adaptive immune cells may not contribute significantly to the pathogenic differences between MIS-C and pCOVID-19. We also observed increased levels of cfDNA derived from megakaryocyte/erythroid progenitors (MEPs) in patients with MIS-C compared with patients with pCOVID-19 or pHCs (Figure 2I; *P* < 0.001).

### Patients with MIS-C have higher end-organ injury than do patients with pCOVID-19.

High levels of cfDNA suggest extensive systemic inflammation, tissue injury, and cell death (32, 33). Consistent with the known clinical presentation of MIS-C involving multiple organ systems (2), we observed higher plasma cfDNA levels derived from nonhematopoietic tissues (Figure 2J; *P* < 0.001) in patients with MIS-C compared with those with pCOVID-19 and pHCs. In particular, we found markedly elevated cfDNA derived from solid organ tissue such as hepatocytes, kidney cells, and cardiac myocytes in cells from patients with MIS-C compared with patients with pCOVID-19 (Figure 2, K–M). In our cohort, 13 of 14 patients with MIS-C had elevated cardiac myocyte cfDNA levels (Supplemental Table 2). The levels of cardiac myocyte–derived cfDNA were 1.2- to 36-fold higher than the median levels for pCOVID-19 (Supplemental Table 3), indicating a varying degree of cardiac involvement in patients with MIS-C. Only 2 of 14 patients with MIS-C in our cohort had elevated gastrointestinal-derived (GI-derived) cfDNA levels (Figure 2N), and 5 of 14 patients with MIS-C had elevated levels of vascular endothelium–derived cfDNA (Figure 2O).

Although lung-derived cfDNA levels were high relative to levels in pHCs, we measured similar injury levels in lung cells from MIS-C and pCOVID-19 (Figure 2P). We also observed increased injury from endocrine systems such as the pancreas and thyroid (Figure 2, Q and R); higher thyroid-derived cfDNA levels were observed in 6 of 14 patients with MIS-C. Presumably reflecting heterogenous interindividual tissue injury, patients with MIS-C had different levels of elevated tissue-specific cfDNA levels. To further assess the potential implication of increased levels of innate immune cell cfDNA in systemic injury, we examined the correlation between innate immune cell cfDNA and tissue-specific or cell-specific cfDNA measurements. Elevated immune cell–derived cfDNA in pediatric patients with or without MIS-C showed a marked correlation with cfDNA derived from solid organs, suggesting a coordinated innate immune response and multiorgan injury (Supplemental Figure 3A).

**Figure 3:**
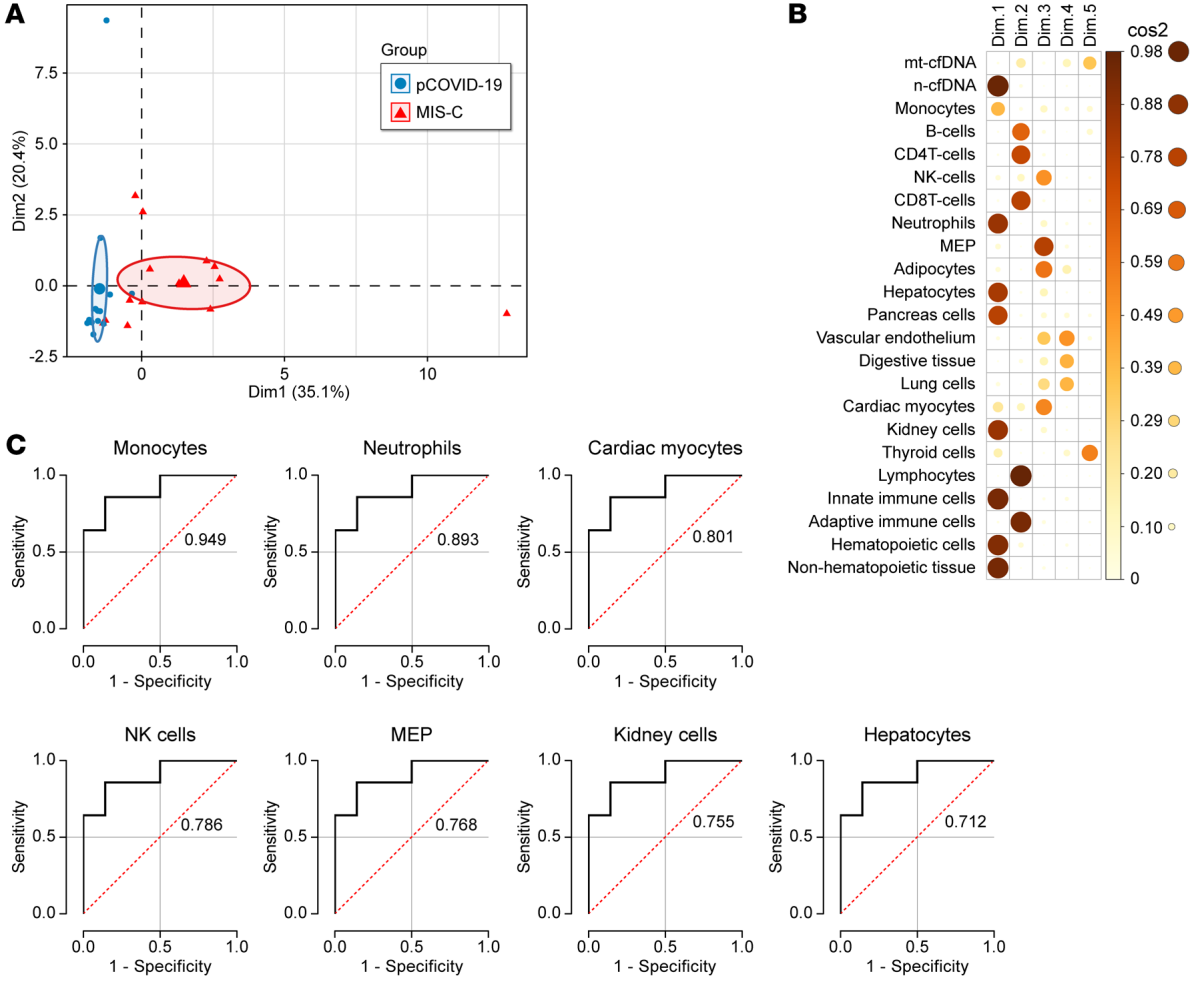
cfDNA profile distinguishes MIS-C and pCOVID-19 with high performance. (**A** and **B**) PCA of cfDNA features to differentiate MIS-C from pCOVID-19. (**A**) Graph representing each patient sample with PC1 (Dim1) on the *x* axis and PC2 (Dim 2) on the *y* axis. The large shapes (red triangle for MIS-C and blue circle for pCOVID-19) represent the average or center of their respective groups, with the ellipses representing 95% CIs for where the true average may lie. The percentages on the axes indicate the amount of variability in the data explained by that axis. (**B**) cos2 plot of the representation for each dimension (Dim) of the PCA. The darker and larger the circle, the more that variable is represented by the dimension it is listed under. The color gradient and size of the circle on the right hand of the panel correlates color with approximate cos2 value. (**C**) ROC curve analysis of tissue-/cell-specific cfDNA measures at admission as a discriminatory marker between pCOVID-19 and MIS-C patients. cfDNA was derived from monocytes, neutrophils, cardiac myocytes, NK cells, MEPs, kidney cells, and hepatocytes.

We next assessed whether tissue-specific cfDNA differences in MIS-C and pCOVID-19 patients were diagnostic classifiers. Principal component analysis (PCA) revealed that cfDNA parameters separated MIS-C from pCOVID-19 by PC1 and PC2, with no overlap in 95% confidence ellipses (Figure 3A). The differences between MIS-C and pCOVID-19 patients were primarily driven by cfDNA originating from combined hematopoietic cells, combined nonhematopoietic tissue, innate immune cells, kidney cells, pancreatic cells, hepatocytes, and neutrophils (Figure 3B). Using ROC analysis (Figure 3C), monocyte-derived cfDNA showed the best discriminatory performance with an AUC of 0.949 (*P* < 0.0001) followed by neutrophils-derived cfDNA (AUC = 0.893, *P* = 0.0006), cardiac myocyte–derived cfDNA (AUC = 0.801, *P* = 0.0058), NK cell–derived cfDNA (AUC = 0.786, *P* = 0.0088), MEP-derived cfDNA (AUC = 0.786, *P* = 0.0108), kidney-derived cfDNA (AUC = 0.755, *P* = 0.0244), and hepatocyte-derived cfDNA (AUC = 0.712, *P* = 0.05). cfDNA derived from lung, the primary target organ of COVID-19 infection, did not discriminate MIS-C from pCOVID-19 (AUC = 0.538, *P* = 0.734). Taken together, the cfDNA profile indicated multisystemic, yet heterogenous, tissue-specific cfDNA elevations, consistent with the clinical presentation of MIS-C. Our data indicate that increased tissue-specific cfDNA levels observed in MIS-C served as accurate classifiers for MIS-C and pCOVID-19.

### Increased cytokine levels in patients with MIS-C compared with levels in patients with pCOVID-19.

**Figure 4:**
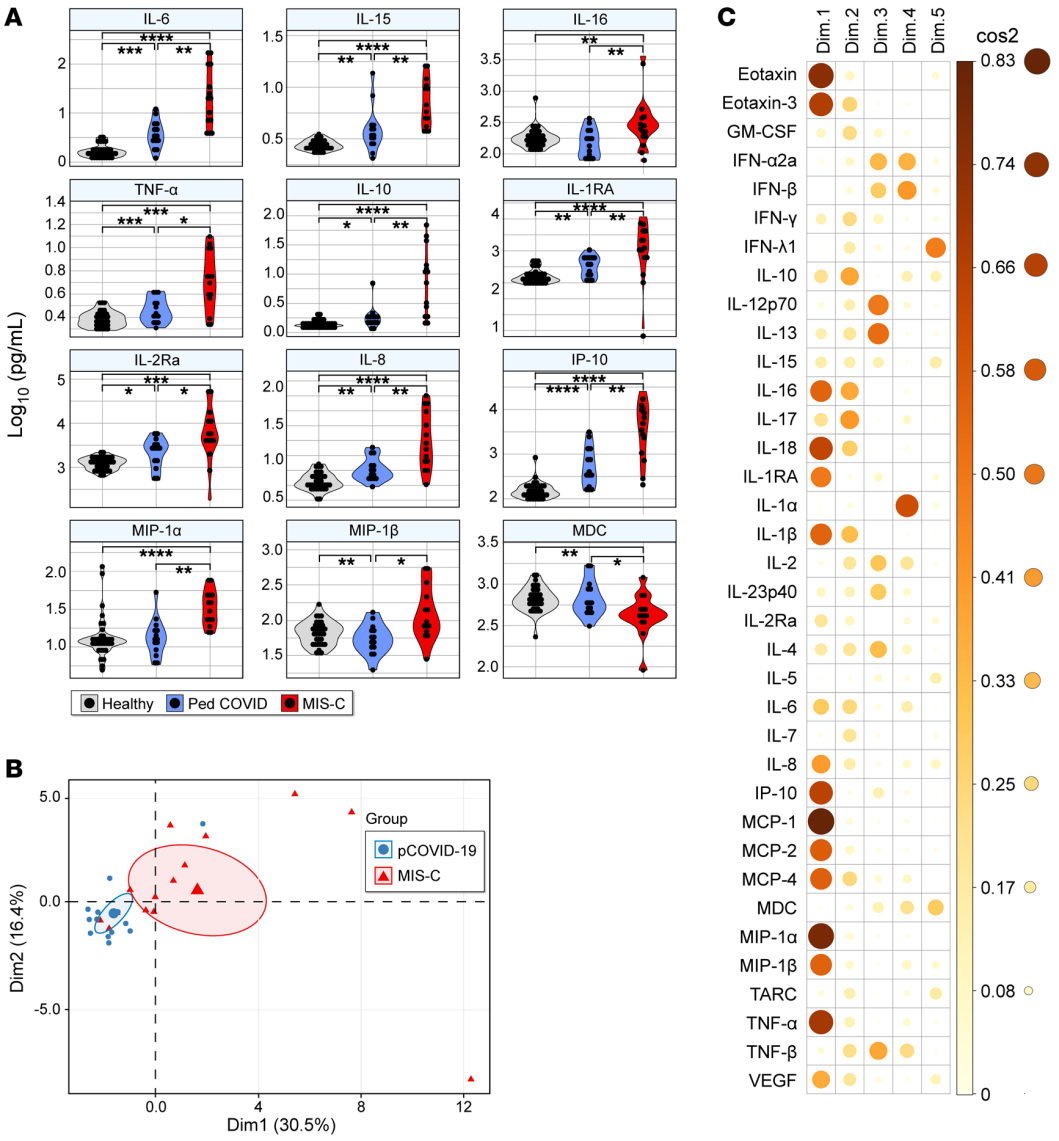
Exaggerated myeloid-derived cytokine levels in MIS-C. (**A**) Comparison of plasma cytokine and chemokine levels in patients with MIS-C, patients with pCOVID-19, and pHCs. Cytokines with a significant difference between MIS-C and pCOVID-19 are shown. Other cytokines are represented in Supplemental Figure 4. Cytokine values are presented as picograms per milliliter (pg/mL; log_10_-transformed). (**B**) Graph of patient sample, with PC1 (Dim1) on the *x* axis and PC2 (Dim2) on the *y* axis. The large shapes (red triangle for MIS-C and blue circle for pCOVID-19) represent the average or center of their respective groups, with the ellipses representing 95% CIs of where the true average may lie. The percentages on the axes indicate the amount of variability in the data explained by that axis. (**C**) Cos2 plot of the representation of each variable for each dimension of the PCA. The darker and larger the circle, the more that variable is represented by the dimension it is listed under. The color gradient on the right hand of the panel correlates color with the approximate cos2 value. *P < 0.05, **P < 0.01, ***P < 0.001, and ****P < 0.0001, by Kruskal-Wallis test followed by Dunn’s multiple comparisons and adjusted for multiple comparison using the Benjamini-Hochberg procedure.

To further investigate the systemic tissue injury/inflammation landscape observed in MIS-C, we measured plasma levels of 36 cytokines and chemokines using a multiplex cytokine assay. A correlation matrix of normalized cytokine levels indicated a strong positive correlation between inflammatory cytokines in all patients (Supplemental Figure 3B). Pairwise comparison identified 11 cytokines and chemokines that were elevated and 1 chemokine, MDC, that was lower in patients with MIS-C compared with those with pCOVID-19 (Figure 4A). The levels of 14 cytokines and chemokines (IL-15, IL-10, IL-13, IL-18, IL-1RA, IL-2Rα, IP-10, IL-6, IL-8, MCP-1, MCP-2, MCP-4, eotaxin, eotaxin-3) were significantly different in both MIS-C and pCOVID-19 patients compared with pHCs (*P* < 0.05 and FDR < 0.1). Compared with pHCs, the levels of 7 cytokines were higher and the levels of 1 cytokine (MDC) were lower in patients with MIS-C. MDC levels were similar in pCOVID-19 patients and pHCs (Figure 4A and Supplemental Figure 4). PCA revealed distinct cytokine signatures for MIS-C and pCOVID-19 (Figure 4B), with a majority of innate immune cytokines being the principal components distinguishing MIS-C and pCOVID-19 (Figure 4C). Cytokines reliably distinguished MIS-C from pCOVID-19 (Supplemental Figure 5). These included IL-6 (AUC = 0.883), MIP-1α (AUC = 0.878), IP-10 (AUC = 0.878), IL-10 (AUC = 0.847), IL-15 (AUC = 0.827), IL-16 (AUC = 0.821), MIP-1β (AUC = 0.821), IL-8 (AUC = 0.821), IL-1RA (AUC = 0.821), TNF-α (AUC = 0.770), IL-2Rα (AUC = 0.750), and MDC (AUC = 0.750). Together, these findings illustrate the shared and distinct pathogenic features of MIS-C and pCOVID-19 related to tissue injury and a dysregulated cytokine response.

### Association of plasma cfDNA with cytokine profiles and laboratory markers.

**Figure 5:**
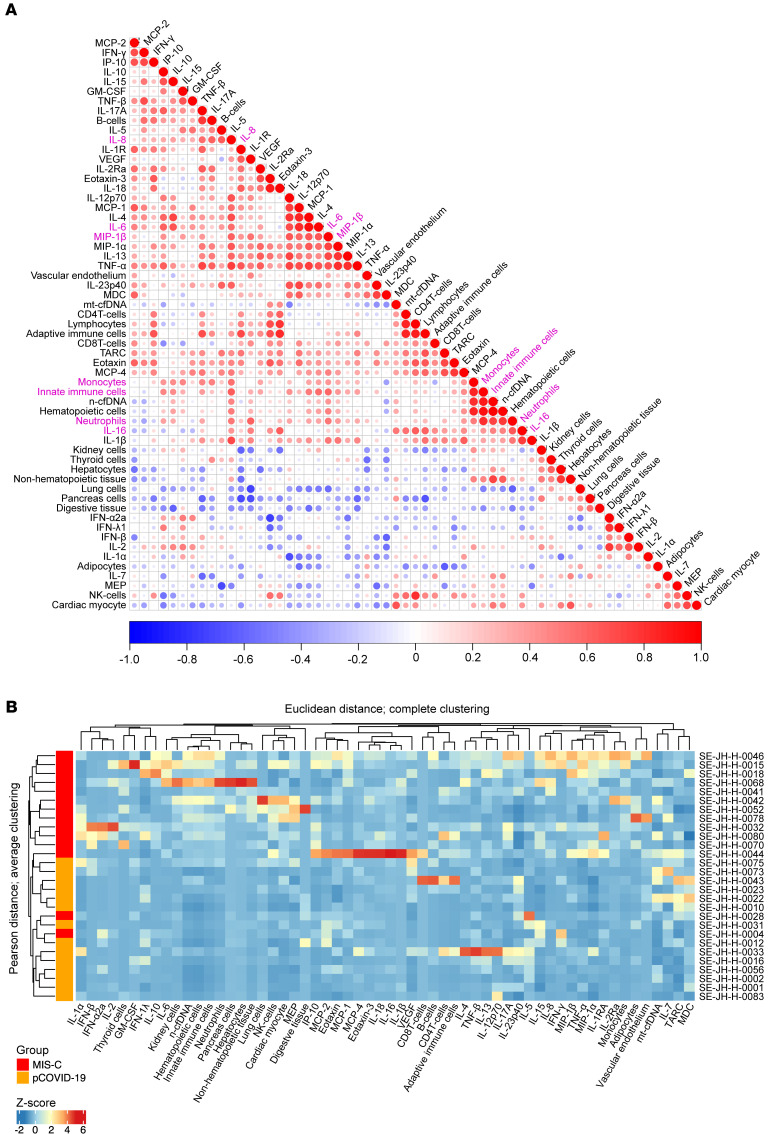
Association of cfDNA levels with the cytokine profile. (**A**) Pearson’s correlation matrix of cfDNA features with cytokine/chemokine profiles in MIS-C. Pink text indicates innate immune cfDNA and cytokines with significant correlations. (**B**) Unsupervised hierarchical clustering heatmap of combined cfDNA and cytokine data for MIS-C and pCOVID-19 patients. Data were scaled and centered (*z* score) for plotting the heatmap using the ComplexHeatmap package (R, version 4.2.1) to normalize the different cfDNA and cytokine scales. Patients dendogram (rows for MIS-C are shown in red and for pCOVID-19 in orange) is based on hierarchical clustering (“average” method) of Pearson’s correlation distances between samples. The feature (column) dendrogram is based on hierarchical clustering (“complete” method) of Euclidean distances between samples/patients.

**Figure 6:**
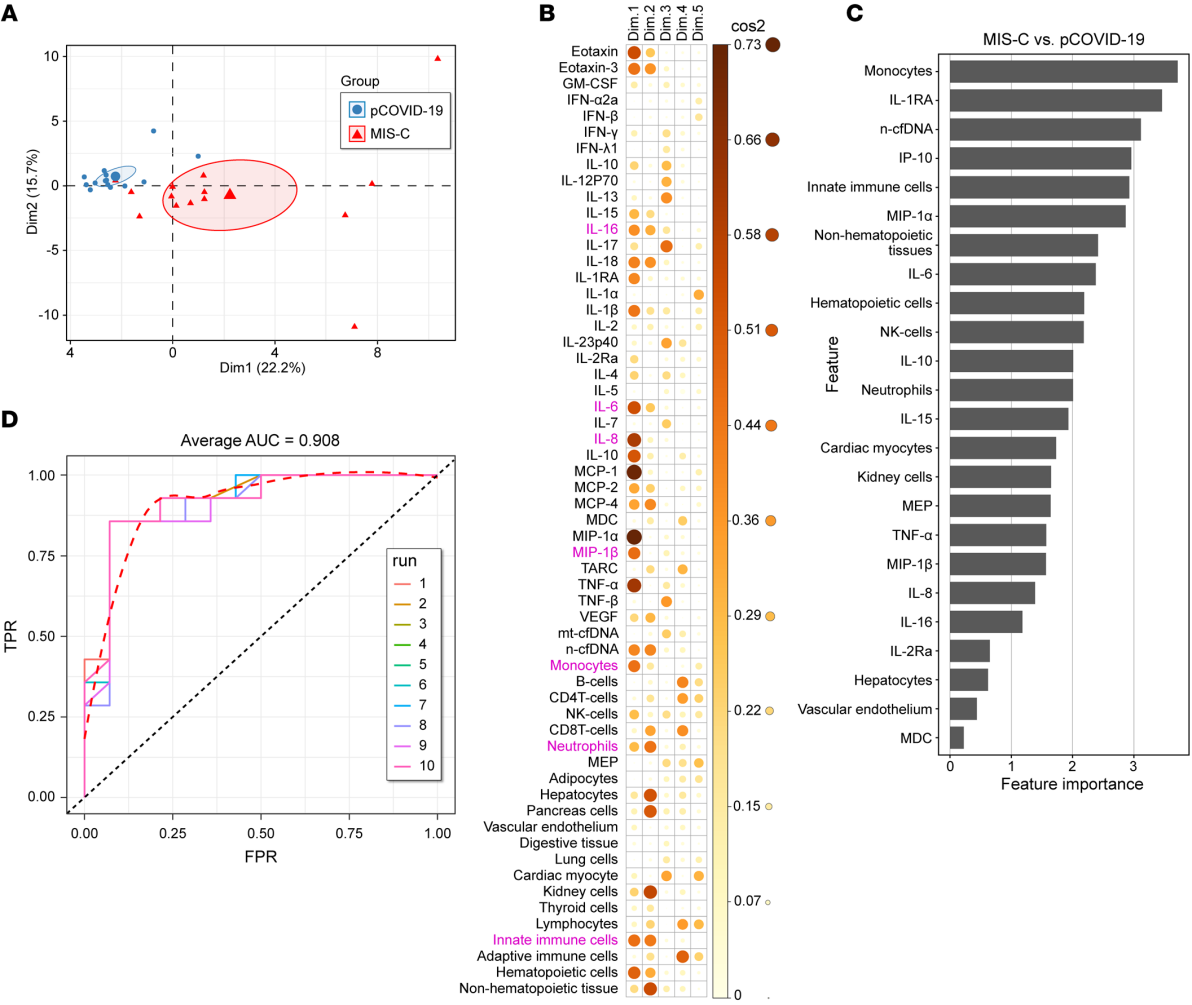
Integrated cfDNA and cytokine analysis distinguishes MIS-C from pCOVID-19. (**A** and **B**) PCA of cfDNA and cytokine profile to differentiate MIS-C from pCOVID-19. (**A**) Graph of each sample with PC1 (Dim 1) on the *x* axis and PC2 (Dim 2) on the *y* axis. The large shapes (red triangle for MIS-C and blue circle for pCOVID-19) represent the average or center of their respective groups, with the ellipses representing 95% CIs of where the true average may lie. The percentages on the axes indicate the amount of variability in the data explained by that axis. (**B**) Cos2 plot of the representation of each variable for each dimension of the PCA. The darker and larger the circle, the more that variable is represented by the dimension it is listed under. The color gradient on the right-hand side of the panel correlates color with the approximate cos2 value. Pink text indicates key innate immune cfDNA and cytokine features. (**C**) Rank of important cfDNA and cytokine features to distinguish patients with MIS-C from those with pCOVID-19 using the random forest model. (**D**) Performance of combined cfDNA and cytokine features to distinguish MIS-C from pCOVID-19. ROC curves for the 10 different runs and the dashed line represent the average curve.

Our correlation analyses identified remarkable correlations between cfDNA and cytokines (Figure 5A). The levels of innate immune cell–derived cfDNA (from neutrophils and monocytes/macrophages) correlated significantly with myeloid-derived inflammatory cytokines (IL-6, IL-8, IL-16 MIP-1β) in patients with MIS-C. Innate immune cytokines also correlated with cfDNA derived from solid organs (Figure 5A). Given the different scales of cfDNA and cytokines, data were scaled and centered to generate *z* scores. Hierarchical clustering of Pearson’s correlation distances between samples/patients demonstrated good separation between pCOVID-19 and MIS-C patients (Figure 5B). PCA of combined cfDNA and cytokine profiles also distinguished pathogenic contributors of MIS-C and pCOVID-19 (Figure 6A), and these differences were mainly driven by myeloid cell–derived cfDNA and cytokines (Figure 6B). Inclusion of the cfDNA and cytokine profiles into a single random forest mode identified the top 24 important cfDNA and cytokine features (Figure 6C) discriminating MIS-C from pCOVID-19, with an AUC of 0.908 (Figure 6D).

We further examined the association between cfDNA levels and conventional laboratory markers of inflammation and organ injury markers in MIS-C (Supplemental Figure 6A). Total and innate immune cell–derived cfDNA measured at the time of hospital admission showed a weak correlation with admission levels of CRP and D-dimer, but a strong positive correlation with markers of disease severity at peak levels of CRP and D-dimer (during the hospital stay, Supplemental Figure 6B). These findings suggest that cfDNA provides an early readout of impending systemic inflammation that manifests later via conventional biochemical markers, indicative of systemic tissue damage. The degree of tissue damage correlated with biochemical measures of tissue injury. For example, hepatocyte-specific cfDNA correlated with liver function tests, including those for alanine aminotransferase (ALT) (*r* = 0.83, *P* = 0.001) and aspartate transaminase (AST) (*r* = 0.65, *P* = 0.003). Two patients with the highest ALT and AST levels also demonstrated the highest hepatocyte-specific cfDNA (Supplemental Table 4). CRP and D-dimer levels demonstrated modest performance in discriminating MIS-C and pCOVID-19 (Supplemental Figure 6C). Using a random forest model (34, 35), we also show that cfDNA measures alone demonstrated performance comparable to that of a model that included combined CRP, D-dimers, and cfDNA measurements (Supplemental Figure 6, C and D). Our findings typify MIS-C as an innate immune–driven hyperinflammatory disease causing interindividual variable multiorgan and tissue injury.

## Discussion

Our findings suggest that cfDNA is an early, sensitive, and noninvasive marker of tissue injury, including that which is clinically occult (36) in MIS-C and pCOVID19, which signals impending pathology in various organs and tissues. Our use of cfDNA as a biomarker for end-organ tissue injury and multiplex plasma cytokine analysis for mapping systemic inflammation infers pathogenic correlates for MIS-C and pCOVID-19. Comprehensive genome-wide cfDNA methylome profiling revealed distinct and exaggerated tissue-specific cfDNA from multiple tissue types in MIS-C compared with pCOVID-19. High but interindividual variable levels of tissue-specific cfDNA among patients with MIS-C confirmed the clinically apparent heterogeneity of this disease and its end-organ involvement. Combined with elevated levels of cfDNA from innate immune origin, cytokine analysis that showed elevated levels of myeloid-derived inflammatory cytokines such as IL-15, IL-16, IL-6, IL-8, TNF-α, MIP-1α, and MIP-1β in MIS-C implicates the innate immune system as a probable driver of MIS-C pathogenesis and suggests potential therapeutic targets.

Somewhat surprisingly, we did not observe a notable correspondence between tissue injury (as measured by cfDNA) in the GI tract or vascular endothelium, 2 commonly affected tissues in patients with MIS-C (37). Literature reports indicate that GI manifestations are common in MIS-C, whereas only 2 of 14 of the patients with MIS-C in our study had high levels of GI-derived cfDNA. However, imaging studies suggest that gastroenteritis, the entity that would be captured by GI-derived cfDNA, may be a less common manifestation of either MIS-C or pCOVID-19 (38, 39). Given the limitations of the pandemic work environment, we did not capture patient symptoms and could not correlate other GI manifestations with cfDNA levels. In addition, the cfDNA algorithm we used may not reliably capture the totality of GI injury because of the many different cell types in this organ. The GI-specific DNA methylation signatures included in our library targets the epithelial cells of the GI tract. Injury to other subregions (e.g., subepithelial and smooth muscle) would be missed. Future experiments aim to tease out these issues.

Although our sample size was comparatively small, we consider it likely that, as with GI cell cfDNA, our DNA methylation library may be inadequate to reliably capture vascular injury in patients with MIS-C and may therefore under-report cfDNA levels of vascular endothelial damage that typically accompanies the thrombotic events commonly observed in both pCOVID-19 and MIS-C. Notably, our previously published cfDNA data in adult patients reliably captured clinical vascular injury and outcomes in adult COVID-19 (9) and adult pulmonary arterial hypertension (3).

The correspondence of baseline levels of cfDNA (at the time of hospital admission) with peak (post-admission) levels of biochemical markers of tissue injury (e.g., CRP and D-dimers) suggest the value of cfDNA as an early marker of tissue injury and systemic inflammation. This pathological pattern is consistent with our prior published findings in the clinical settings of heart or lung transplantation, wherein the levels of allograft-derived cfDNA rise well before detection by physical damage (biopsy), enabling a noninvasive, sensitive predictor of acute rejection and its severity.

Hematopoietic cells and solid organs (heart, kidney, and liver) were the major contributors of the high cfDNA in both pCOVID-19 and MIS-C. However, compared with patients with pCOVID-19, patients with MIS-C had higher levels of total and tissue-specific cfDNA from hematopoietic tissues and solid organs (heart and liver). In addition, MIS-C, but not pCOVID-19, showed higher levels of cfDNA from kidney and, surprisingly, from endocrine organs. These cfDNA tissue sources are consistent with reports of the myocardial injury, hepatitis, and acute kidney injury observed patients with MIS-C (37, 40, 41). Our findings are consistent with proteomics studies showing increased cardiac-specific antigens in MIS-C (42). Our analysis revealed substantially higher levels of cardiac-derived cfDNA in MIS-C compared with pCOVID-19.

Our findings are consistent with innate immunity as the predominant driver of the systemic inflammation observed in MIS-C as compared with pCOVID-19. Higher cfDNA from endocrine organs found in a fraction of patients with MIS-C is consistent with observations of thyroid and adrenal insufficiency in some patients with MIS-C (43, 44). In our study, patients with MIS-C also had increased levels of circulating cfDNA derived from megakaryocyte-erythroid precursor cells compared with patients with pCOVID-19. These cells were the third major contributor to total plasma cfDNA (45). Megakaryocytes are implicated in immunothrombosis, a common complication in MIS-C (46). Single-cell RNA-Seq (Gene Expression Omnibus [GEO] GSE166489) (47) analysis showed that these same cell types upregulate programmed cell death pathway genes, apoptosis, necroptosis, and pyroptosis in patients with MIS-C compared with pHCs. The adaptive immune cell compartment revealed comparable T cell– and B cell–derived cfDNA in MIS-C and pCOVID-19 patients. Immunoregulatory cytokines (such as MDC) that promote an adaptive regulatory T cell response (48) were significantly lower in patients with MIS-C compared with patients with pCOVID-19 and pHCs. While MIS-C and pCOVID-19 patients had similar absolute counts of circulating neutrophils, we observed elevated cfDNA derived from innate immune cells, including neutrophils, monocytes/macrophages, and NK cells predominantly in patients with MIS-C compared with patients with pCOVID-19, in agreement with published work implicating neutrophils and monocytes/macrophages as central players in MIS-C pathogenesis (16, 49).

Because MIS-C is a serious acute syndrome that requires a prompt response, a fast, sensitive, and reliable biomarker of early tissue injury is of great clinical value. Given the heterogeneous clinical presentation of MIS-C, a profile of end-organ involvement in each patient, including occult tissue types, may guide an individualized treatment strategy. The integrated cfDNA and cytokine analysis we have described here has broad clinical and mechanistic implications. As noted, an accurate, noninvasive marker of early injury offers advantages over conventional inflammatory and organ injury markers that may appear later in the clinical course of pCOVID-19 and MIS-C. The cfDNA-based epigenetics analysis we used in this study enables early characterization of relevant tissue injury in multiple disease settings (3–6), including injury from remote tissue types (12). For comparison, in solid organ transplantation settings, plasma allograft–derived cfDNA levels detect rejection 2–4 months earlier than do histopathological, echocardiographic, or clinical manifestations (4, 50). Going forward, the analysis of cfDNA epigenetic landscapes across tissue types could inform biological pathways related to disease pathology (13). In adult COVID-19, we showed that plasma cfDNA contributes to an oxidative environment in a concentration- and time-dependent manner (9). The release of cfDNA from injured cells is associated with the formation of NETs, which have been shown to be elevated in COVID-19 and contribute to immunothrombosis and organ damage (51).

Our study has limitations. Our findings are limited by a small sample size due to the rarity of MIS-C, even at a major metropolitan children’s hospital. Second, as discussed above, our cfDNA methylation algorithm does not include all cell types of the human body, and our deconvolution analysis was restricted to the detection of major cell types of the tissue or organ of origin. Because of the pandemic work conditions, we were only able to collect patient blood samples at the time of hospital admission, limiting the ability to analyze time courses of plasma cfDNA and cytokine production. Although we identified a strong correlation between cfDNA and circulating cytokines, our analyses and small sample size preclude the identification of a directional mechanistic link. Future mechanistic studies elucidating causal relationships between cfDNA and inflammatory pathways may illuminate therapeutic strategies. Nonetheless, to our knowledge, this study is among the first to use cfDNA tissue profiling to infer disease pathogenesis. Our results also shed light on potential endotypes of MIS-C, offering mechanistic clues toward developing precision treatment not only for MIS-C, but also for multiorgan inflammatory conditions of childhood such as Kawasaki disease and others.

## Methods

### Study design and participants.

Twenty-eight pediatric patients (≤18 years of age) with COVID-19 infection confirmed by a PCR test for SARS-COV-2 admitted to Johns Hopkins Hospital (JHU) between March 2020 and March 2021 were included in this study; 14 met the criteria for MIS-C according to the CDC (52), and 14 were patients with acute pCOVID-19 with no evidence of MIS-C. Demographic, clinical, and laboratory data were extracted from the patients’ medical charts. The primary goal of this study was to define tissue injury patterns using circulating cfDNA and to profile circulating cytokine levels in patients with MIS-C and acute pCOVID-19. The secondary objectives were to assess the correlation between tissue cfDNA patterns with circulating cytokines and chemokines and conventional clinical markers. An overview of study participants and experimental workflow is shown in Figure 1A.

### Sample collection.

Peripheral blood samples were collected into EDTA vacutainer tubes at the time of hospital admission before infusion of IVIG and biologics. Plasma samples were collected by centrifuging at 1,600*g* for 10 minutes at 4°C, aliquoted to 1 mL volume in Eppendorf tubes, and stored at –80°C until use. Plasma was thawed and then centrifuged at high speed (16,000*g*) for 10 minutes at 4°C to remove residual debris. Plasma was spiked with fragmented unmethylated lambda DNA (725 copies/mL) to calculate extraction efficiency and the bisulfite conversion rate. Then, plasma cfDNA was isolated on an automated nucleic acid sample preparation QIAsymphony^SP^ (QIAGEN) instrument using the QIAsymphony DSP Circulating DNA kit according to the manufacturer’s protocol. The extracted cfDNA was eluted in 60 μL in LoTE buffer, quality checked using the Cell-free DNA ScreenTape assay on the 4150 TapeStation System (Agilent Technologies), and stored at –20°C for further use.

### cfDNA quantification.

The absolute copy number of cell-free nuclear and mitochondrial DNA was measured as described earlier using a droplet digital PCR (ddPCR) system (53). Briefly, a total of 22 μL reaction mix was prepared, in triplicate, by adding 11 μL 2× ddPCR Supermix for Probes (no deoxyuridine triphosphate [dUTPs]), 0.50 μL 20× FAM-labeled ddPCR assay, 0.50 μL HEX-labeled ddPCR assay, 6 μL nuclease-free water, and 4 μL template cfDNA (diluted 1:10). The PCR reaction was thoroughly mixed, and droplets were generated using the QX200 Droplet Generator, followed by thermal cycling (1 cycle at 95°C for 10 min, 40 cycles [ramp rate 2.5°C/s] at 94°C for 30 s, 60°C for 1 min, and then 98°C for 10 min). Four different primers/probes (AP3B1, TERT, AGO1, and RPP30) for n-cfDNA, 1 (ND1) for mt-cfDNA and 1 for lambda were used (Bio-Rad). Samples were read with QX200 Droplet Reader with QuantaSoft Software and analyzed using QuantaSoft Analysis Pro software. The extraction efficiency of cfDNA was calculated by dividing the absolute copy number of lambda DNA recovered by the expected plasma spiked-in lambda DNA value. The levels of n-cfDNA (average of 4 targets) and mt-cfDNA in plasma were expressed as copies per milliliter plasma sample (cp/mL plasma) after adjusting for the dilution factor, plasma volume used, and extraction efficiency.

### cfDNA library preparation and sequencing analysis.

The extracted cfDNA underwent bisulfite treatment (EZ DNA Methylation-Gold Kit, Zymo Research) according to the manufacturer’s recommendations. Sequencing libraries were constructed using the Accel-NGS Methyl-Seq DNA Library Kit with Unique Dual Indexing (Swift Biosciences) for whole-genome bisulfite sequencing according to the manufacturer’s instructions. Libraries were quality checked with high-sensitivity D1000 ScreenTape (Agilent Technologies), quantified with the Quant-iT PicoGreen dsDNA assay kit (Life Technologies, Thermo Fisher Scientific), pooled with an equimolar concentration on an epMotion 5070 instrument, and sequenced on an Illumina NovaSeq 6000 machine using 2 × 100 bp reads. The raw sequencing reads were quality checked with FastQC (54), version 0.11.9, trimmed while retaining paired-end reads with a minimum length of 50 bp using TrimGalore (55), version 0.6.7, and mapped to the bisulfite-converted human reference genome (version hg19) with Bismark (56), version 0.23.0, using Bowtie2, version 2.4.5, as the default aligner. Bismark was also used to remove PCR duplicates and extract cytosine methylation (CpG) states in all individual samples. The average mapping efficiency, deduplication rate, base coverage, and sequencing depth were 88.7% ± 0.8%, 89.53% ± 4.21%, 5% ± 0.76%, and 5.63% ± 0.84%, respectively. The efficiency of bisulfite conversion was determined using the spiked-in lambda DNA and resulted in an average conversion rate of 99.95% ± 0.004%. The cell or tissue origin of cfDNA was deconvoluted using human cell or tissue-type–specific methylation signatures as a reference with meth_atlas algorithm (12). To obtain absolute concentrations, the estimated proportions of cell- or tissue-specific cfDNA were multiplied by the total concentration of nucleus-derived cfDNA (copies/mL) in plasma. The deconvolution plots were generated using R software, version 4.2.2.

### Multiplex cytokine/chemokine measurement.

Plasma samples from 14 patients with MIS-C, 14 patients with pCOVID-19, and 35 pHCs were measured, in duplicate, for 36 different cytokines (IFN-γ, IFN-α2a, IFN-β, IFN-λ1, IP-10, IL-10, IL-12p70, IL-13, IL-15, IL-16, IL-17A, IL-1α, IL-RA, IL-1β, IL-2, IL-2Rα, IL-23p40, IL-4, IL5, IL-6, IL-7, TNF-α, TGF-β GM-CSF and VEGF) and chemokines (IL-8, MIP-1α, MIP-1β, MCP-1, MCP-2, MCP-4, eotaxin, eotaxin 3, MDC, TARC) using the multiplex assay kit from Meso Scale Discovery (MSD) according to the manufacturer’s instructions. Data were acquired with a MESO QuickPlex SQ 120 instrument, and if the cytokine values were below the background, the values were set to 0.

### Collection and analysis of cell death gene transcripts.

Publicly available single-cell RNA-Seq data from peripheral blood cells of patients with MIS-C and pHCs were downloaded from the NCBI’s GEO database (GEO GSE166489). We used the processed data and the annotation provided in ref. 47 to obtain the gene expression of myeloid cells, neutrophils, B cells, T cells, and NK cells. The counts for each gene were aggregated within each cell type across the cells to form a pseudobulk sample for each cell type from each patient. Then, pseudobulk counts were normalized using the NormalizeData function in the Seurat R package (57). To remove the batch effect, the sva function in the sva R package (58) was used to estimate and remove the effect of surrogate variables from the pseudobulk samples. Gene expression of the cell death–related genes was obtained from the pseudobulk sample of each cell type from each patient. To compare gene expression between patients with MIS-C and pHCs, a 2-sided *t* test was applied to each gene within each cell type. *P* values were transformed to the FDR to adjust for multiple testing using the Benjamini-Hochberg procedure (59).

### Statistics.

Data are presented as frequencies (proportions) for categorical variables and as the median (IQR) continuous variables. Fisher’s exact or χ^2^ test was used to compare categorical variables between groups. Comparisons between 3 groups of continuous variables were conducted using a Kruskal-Wallis test with Dunn’s correction for multiple comparisons. A Mann-Whitney *U* test was used for comparison of 2 continuous group variables unless otherwise stated. ROC curve analysis was performed to determine the discriminating performance of cfDNA and the cytokine profile in children with or without MIS-C. The random forest model was applied to evaluate the discriminative performance of combined cfDNA and cytokine features using the leave-one-out cross-validation approach. The training and testing process was repeated 10 times for the data, which was log_2_(× + 1) transformed. The AUC ROC is the average value of the 10 different runs. The relative importance for each the cfDNA and cytokine features was assessed using the mean increase in error rate (decrease in accuracy) over all out-of-bag cross-validated predictions. Pearson’s correlation coefficient was used to examine the correlations between cfDNA levels with cytokine profiles and clinical characteristics. PCA was carried out using the R packages FactoMineR and factoextra to estimate the relative contribution of cfDNA and cytokine data for separation between groups. Unsupervised hierarchical clustering analysis was performed to create a heatmap using ComplexHeatmap R package. A *P* value less than 0.05 was considered statistically significant. GraphPad Prism (version 9.4.1) and R (version 4.2.1) software was used for statistical analysis and generation of graphs.

### Study approval.

This study was approved by the IRBs of the Johns Hopkins University. For an additional comparison, we included plasma samples obtained in the morning at outpatient visits from 35 pHCs under a protocol (NCT02179151) approved by the NIH Clinical Center IRB. Written informed assent and consent was obtained from all participants or their legal guardians and conducted according to the Declaration of Helsinki.

### Data availability.

The data set used in this manuscript is available in the supplemental material in a single Excel (XLS) file named Supplemental Supporting Data Values. All the values for all data points shown in the graphs are displayed in a separate tab. The whole-genome bisulfite sequencing data are not publicly deposited because of privacy/ethics restrictions and are available from the corresponding author upon reasonable request. For code used in this study, the methylation analysis scripts are provided on Zenodo (https://doi.org/10.5281/zenodo.8387344).

## Author contributions

TEA and SAE conceived and designed the experiments. TEA, KR, HK, WP, RM, TSJ, and MKJ performed experiments. TEA wrote the manuscript draft. NR, TH, ZA, WZ, and YW conducted bioinformatics analysis. TEA, KR, WZ, and AHK performed statistical analyses. OG, SB, JAY, and ALC were involved in recruiting study participants and sample collection. HK performed statistical analyses. CML helped supervise the project. SAE supervised the study. All authors participated in the preparation of the manuscript and gave final approval for publication.

## Supplementary Material

Supplemental data

Supplemental Acknowledgments

Supporting data values

## Figures and Tables

**Table 1:**
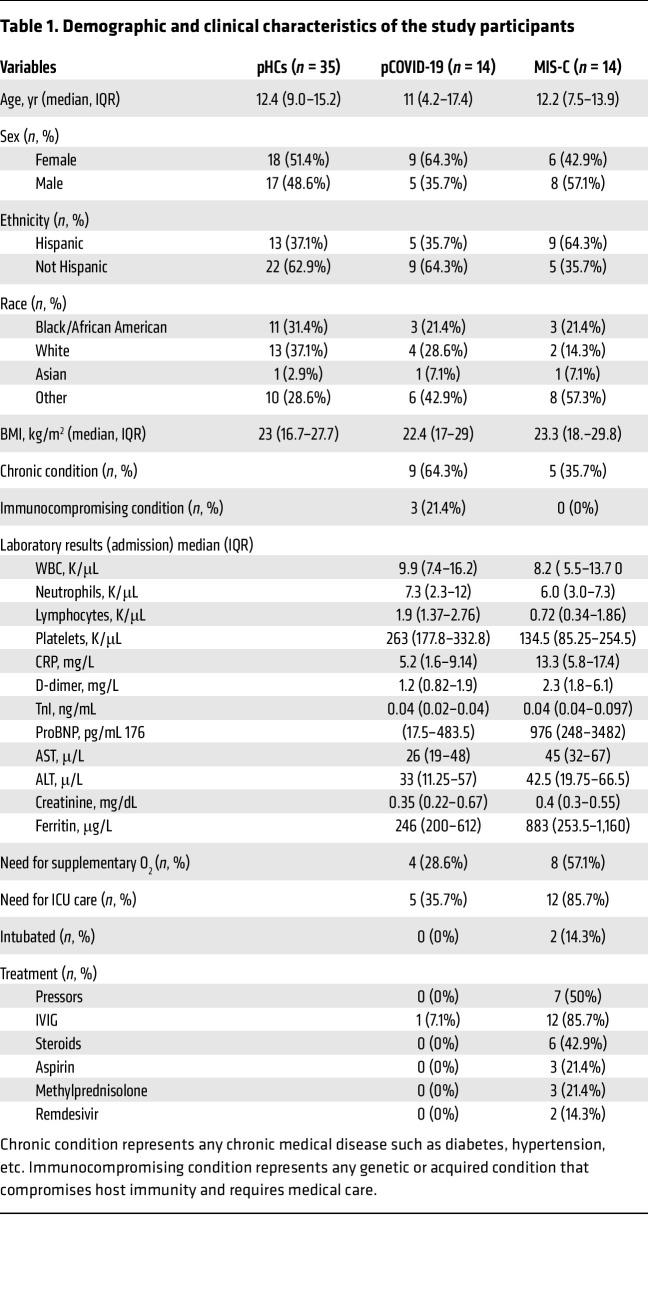
Demographic and clinical characteristics of the study participants

## Version 1 08/31/2023

In-Press Preview

## Version 2 11/01/2023

Electronic publication
