# Supplementary material for: Cell-free DNA reveals distinct pathology of multisystem inflammatory syndrome in children
Source: J Clin Invest. 2023 Nov 1;133(21):e171729. doi: 10.1172/JCI171729 (PMC10617770; doi:10.1172/JCI171729)
Supplement: Supplemental data [file jci-133-171729-s215.pdf]

Supplemental Materials

Cell-death specific gene expression in MIS-C

To gain insight into the potential mechanisms of cell death, we obtained publicly available single-cell RNA sequencing transcriptome dataset (GSE166489) of a different cohort of MIS-C patients (47). We surveyed gene transcripts related to cellular death pathways in immune cells of MIS-C patients and compared the gene transcripts to healthy pediatric controls (HCs) from public dataset. Supporting our cfDNA results, we found significantly upregulation of cell-death pathways in MIS-C compared to HCs (Suppl. Fig. 7). In the scRNAseq analysis dataset adapted from Ramaswarmy et al., neutrophils in MIS-C patients clustered into two distinct groups. Cluster 1 neutrophils showed significantly more cell death than neutrophils in cluster 2 (Suppl. Fig. 7A&B). Myeloperoxidase (MPO), peptidyl arginine deaminases (PADI2, PADI4), azurocidin 1 (AZU1) and neutrophil elastase (ELANE) that contribute to neutrophil degranulation and a pro-inflammatory cell death unique to neutrophils called NETosis were highly upregulated in MIS-C patients, supporting our finding of an increased neutrophil-derived cfDNA (Fig 2C). Cluster 1 neutrophils (Suppl. Fig. 7A) in MIS-C also upregulated genes involved in the apoptotic cell death pathway (FOXO3, BAX, and CYCS). Gasdermin D (GSDMD), an important player in the pyroptotic cell death was significantly upregulated in MIS-C compared to HCs in neutrophil cluster 1 (Suppl. Fig. 7A). Genes involved in autophagy (DRAM1, BECN1) and necroptosis (MLKL, RIPK1, RIPK3) were not upregulated in MIS-C neutrophils, and neither were most genes involved in ferroptosis (FANCD2, GPX4, and IREB2) apart from ALOX15B, a gene known to localize in the cell membrane during the iron-dependent cell death (Suppl. Fig. 7A&B). ALOX15B was highly upregulated in both

neutrophil clusters in MIS-C patients compared to HCs (Suppl. Fig. 7A&B). Additionally, monocytes and dendritic cells, exhibited marked upregulation of transcripts involved in pyroptosis (GSDMD, PYCARD), apoptosis (BAX, CYCS, AIFM1, FAS) and necroptosis (MLKL, RIPK1, RIPK3) (Suppl. Fig. 7C). This is consistent with our cfDNA results suggesting that myeloid cells significantly contribute to plasma cfDNA levels in MIS-C patients. T cells, B cells and NK cells exhibited no difference in production of transcripts involved in cell death pathways between MIS-C and HC (Suppl. Fig. 7D-F), consistent with our findings that cells of lymphoid cell lineage contribute less to plasma levels of cfDNA in MIS-C patients. We also observed upregulation of the pro-apoptotic gene CYCS in MIS-C B cells, but significant decrease of CASP9 (apoptosis) and RIPK1 (necroptosis) compared to HCs (Suppl. Fig. 7E). Overall, the increased gene transcripts related to cellular death pathways support the excessive cfDNA release in the circulation of MIS-C patients.

A

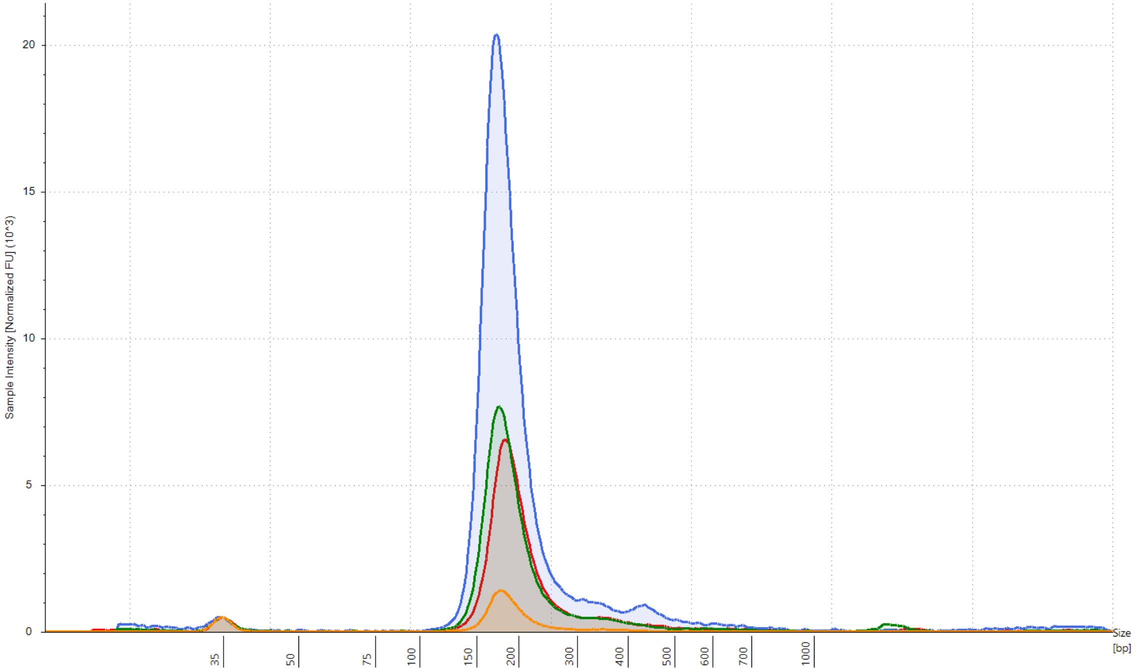
Sample Intensity [Normalized FU] (10^3)
20
15
10
5
0
35
50
75
100
150
200
300
400
500
600
700
1000
Size
[bp]

B

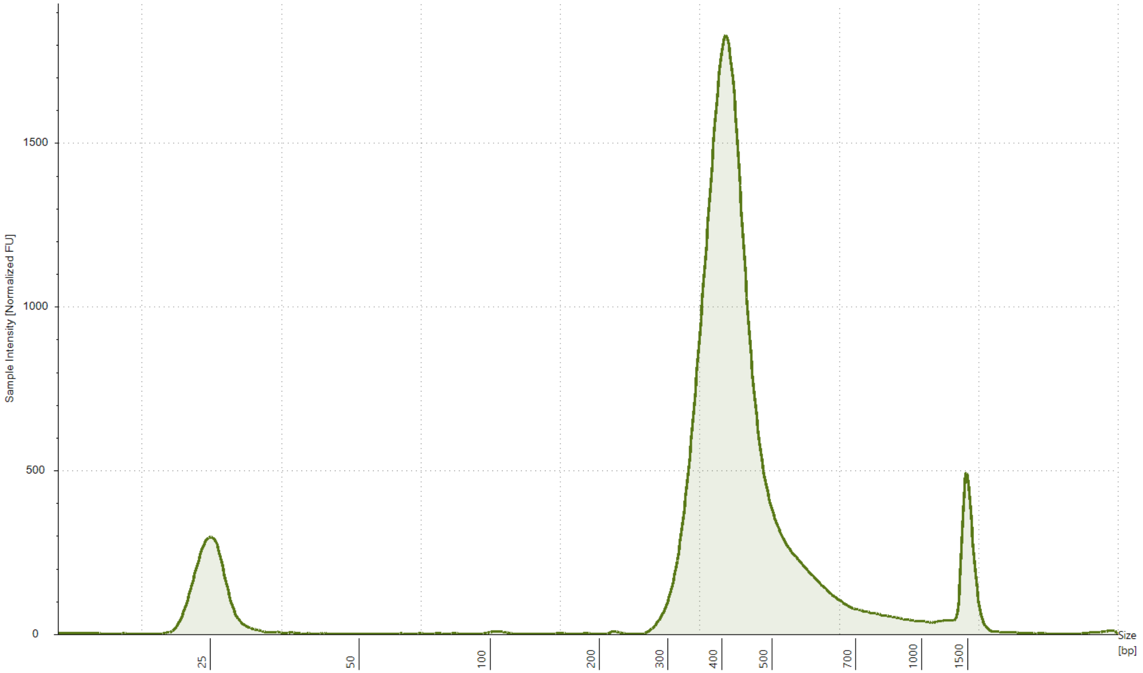
Sample Intensity [Normalized FU]
1500
1000
500
0
25
50
100
200
300
400
500
700
1000
1500
Size
[bp]

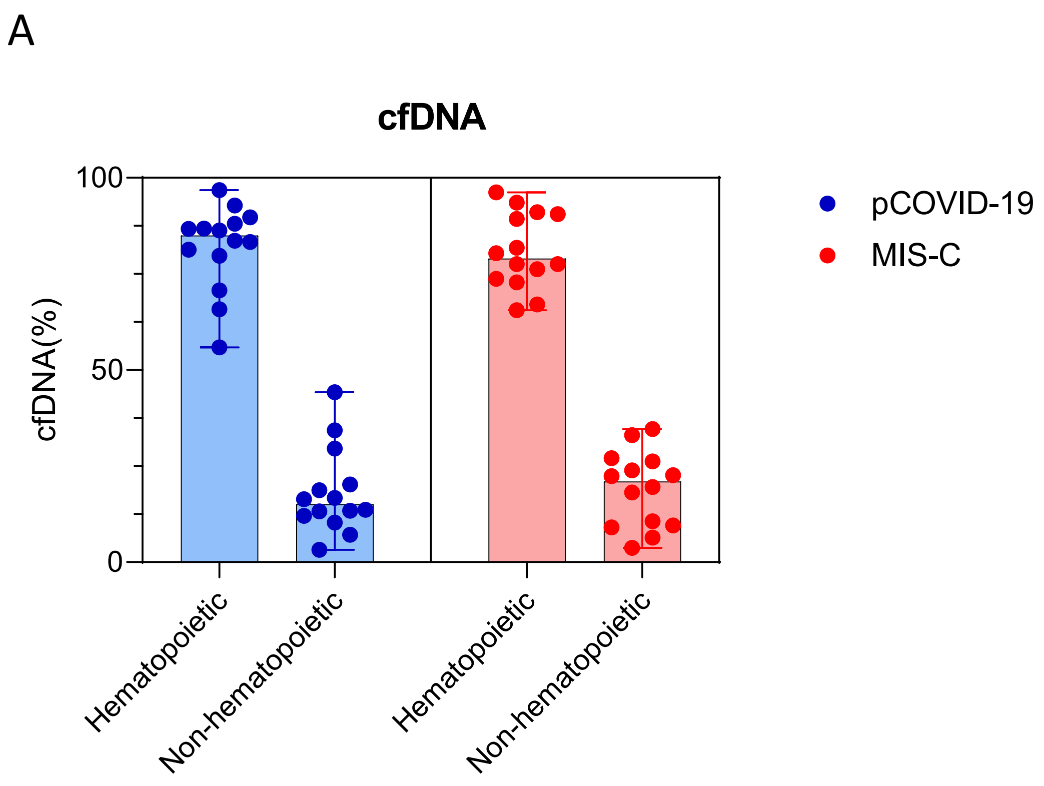

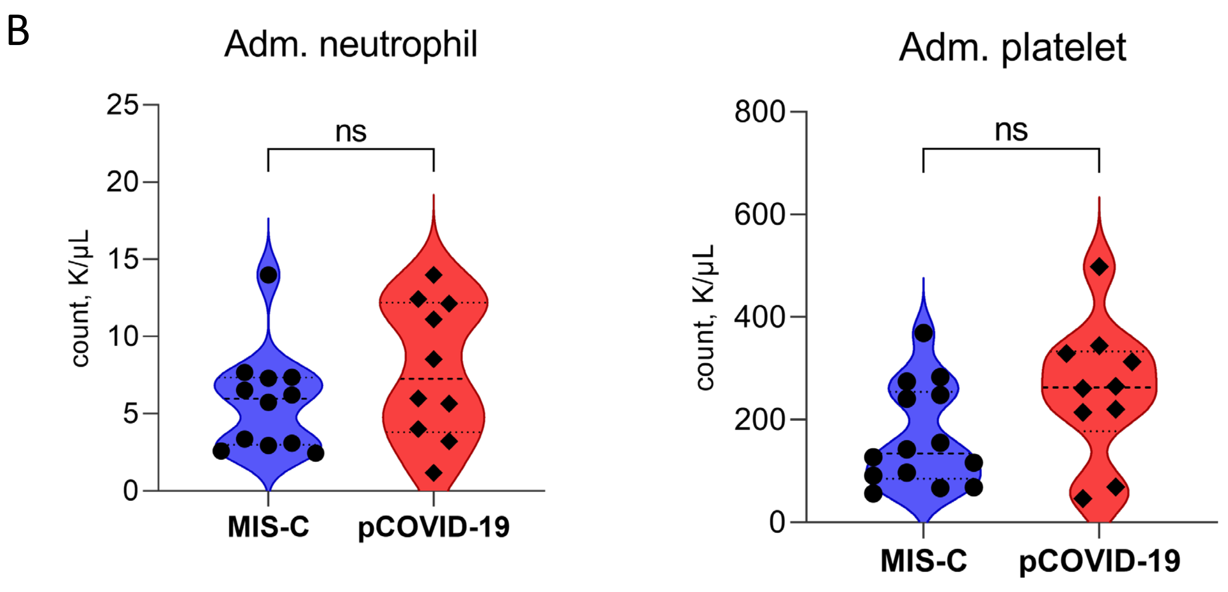

	MIS-C (median [IQR])	pCOVID-19 (median [IQR])
Neutrophil, K/μL	5.97 (2.98 (5.97 -7.34)	7.26 (3.8 - 12.2)
Platelet, K/μL	134.5 (85.2 - 254.5)	263 (177.8 - 33.8)

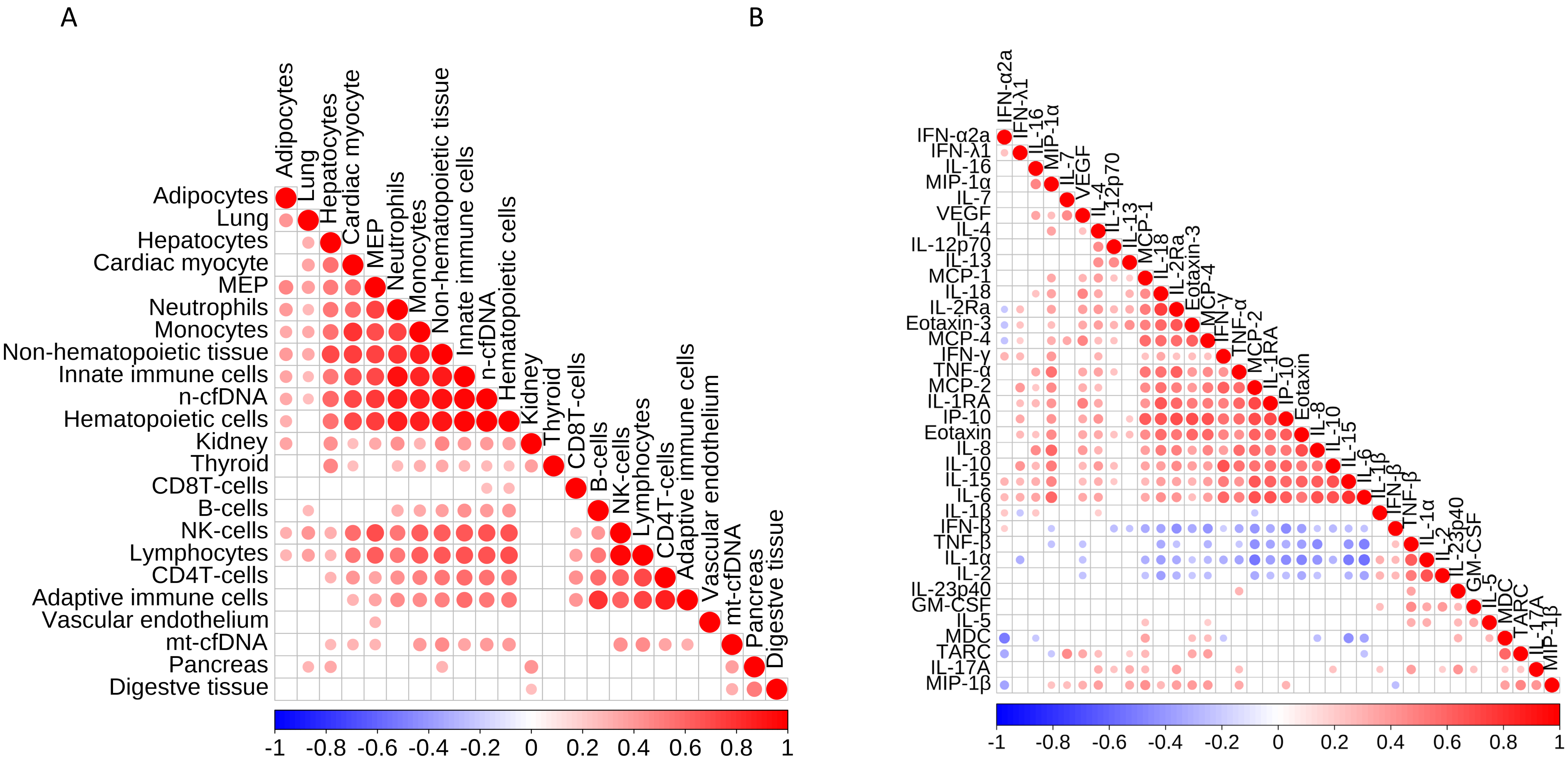
A
B
Adipocytes
Lung
Hepatocytes
Cardiac myocyte
MEP
Neutrophils
Monocytes
Non-hematopoietic tissue
Innate immune cells
n-cfDNA
Hematopoietic cells
Kidney
Thyroid
CD8T-cells
B-cells
NK-cells
Lymphocytes
CD4T-cells
Adaptive immune cells
Vascular endothelium
mt-cfDNA
Pancreas
Digestve tissue
-1 -0.8 -0.6 -0.4 -0.2 0 0.2 0.4 0.6 0.8 1
IFN-α2a
IFN-λ1
IL-16
MIP-1α
IL-7
VEGF
IL-4
IL-12p70
IL-13
MCP-1
IL-18
IL-2Ra
Eotaxin-3
MCP-4
IFN-γ
TNF-α
MCP-2
IL-1RA
IP-10
Eotaxin
IL-8
IL-10
IL-15
IL-6
IL-1β
IFN-β
TNF-β
IL-1α
IL-2
IL-23p40
GM-CSF
IL-5
MDC
TARC
IL-17A
MIP-1β
-1 -0.8 -0.6 -0.4 -0.2 0 0.2 0.4 0.6 0.8 1

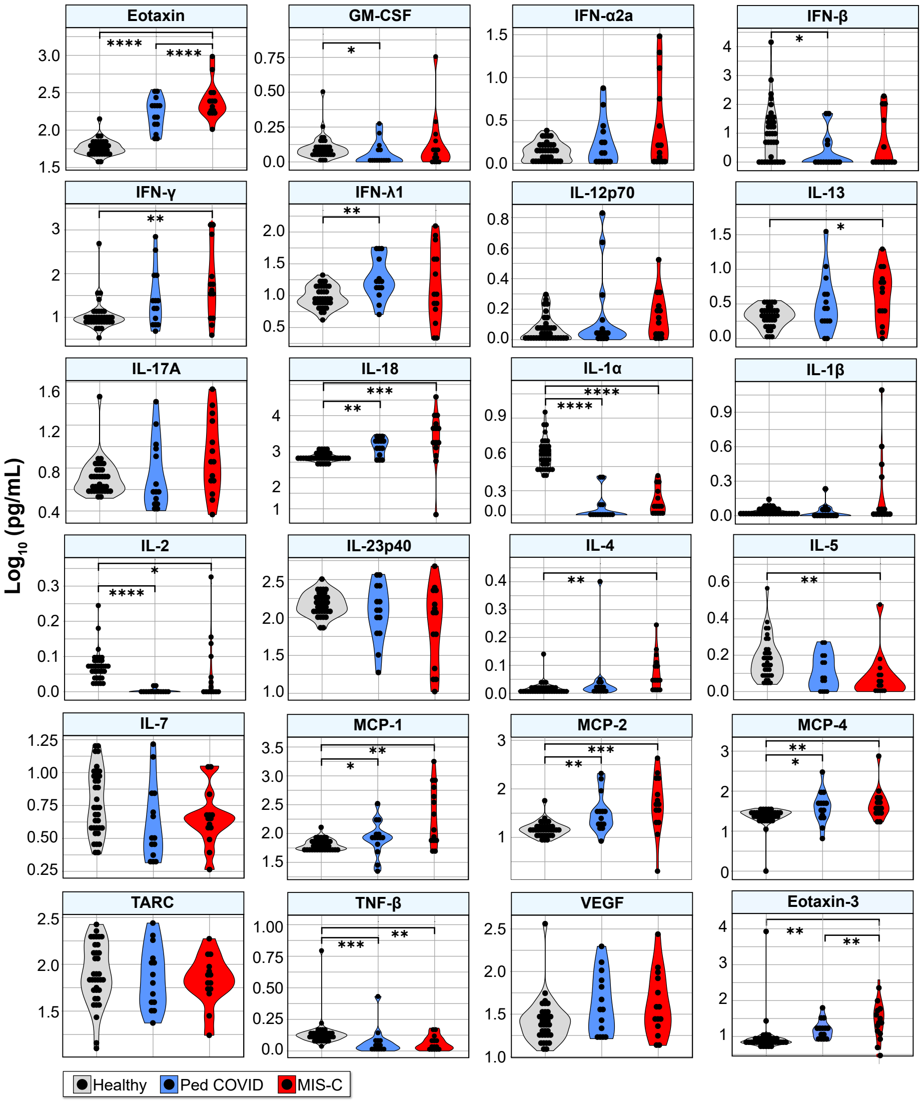

Eotaxin
GM-CSF
IFN-α2a
IFN-β
IFN-γ
IFN-λ1
IL-12p70
IL-13
IL-17A
IL-18
IL-1α
IL-1β
IL-2
IL-23p40
IL-4
IL-5
IL-7
MCP-1
MCP-2
MCP-4
TARC
TNF-β
VEGF
Eotaxin-3
Log10 (pg/mL)
Healthy
Ped COVID
MIS-C

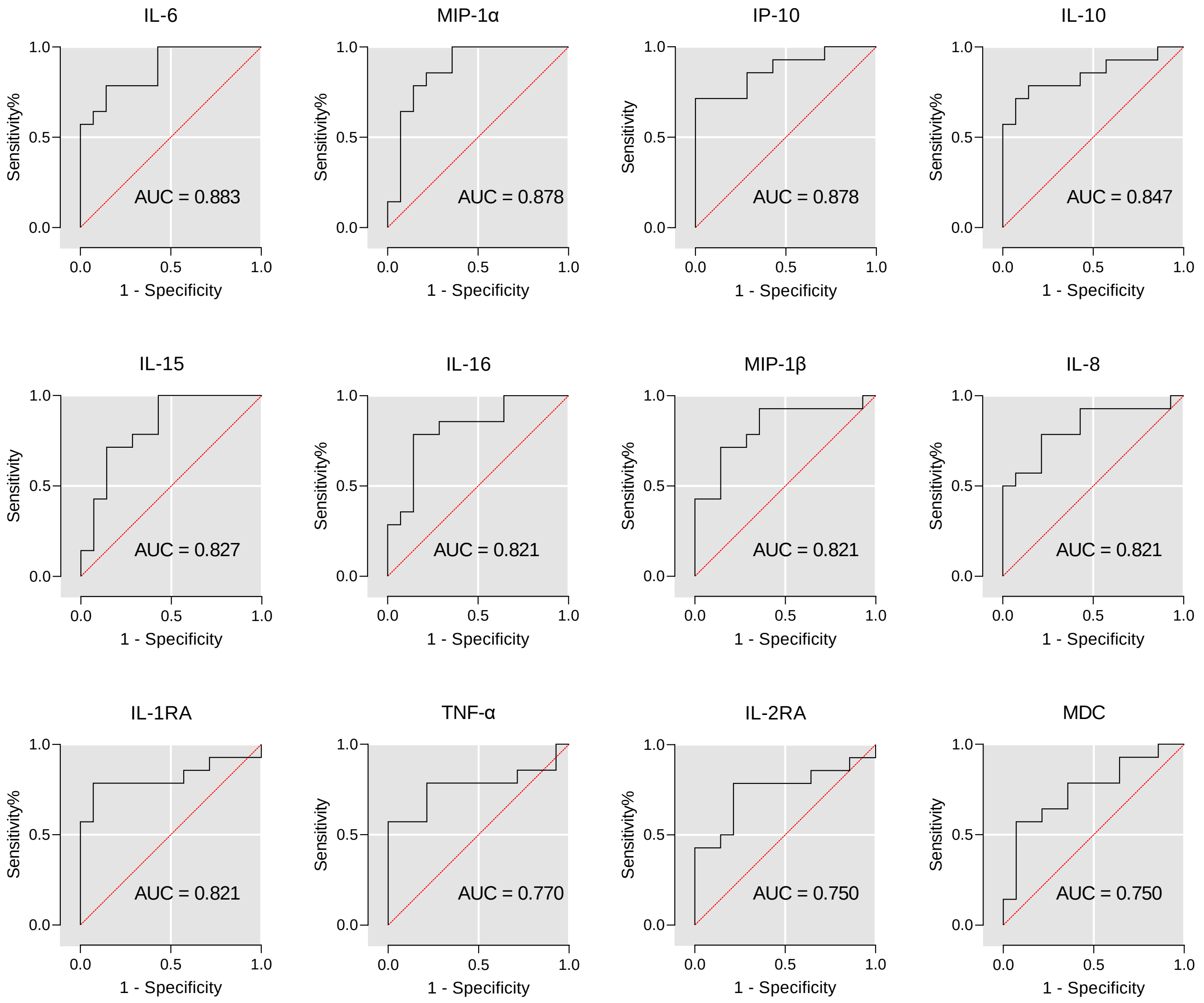

Cytokines	IL-6	MIP-**1α**	IP-10	IL-10	IL-15	IL-16	MIP-**1β**	IL-8	IL-1RA	TNF-**α**	IL-**2Rα**	MDC
AUC	0.883	0.878	0.878	0.847	0.827	0.821	0.821	0.821	0.821	0.77	0.75	0.75
95% CI	[0.761 – 1.0]	[0.741 – 1.0]	[0.747 – 1.0]	[0.695 - 0.999]	[0.669 - 0.984]	[0.661 - 0.982]	[0.659 - 0.984]	[0.660 - 0.983]	[0.646 - 0.996]	[0.579 - 0.962]	[0.557 - 0.943]	0.564 - 0.936
p-value	0.0006	0.0007	0.0007	0.0018	0.0033	0.0038	0.0038	0.0038	0.0038	0.0149	0.0244	0.0244

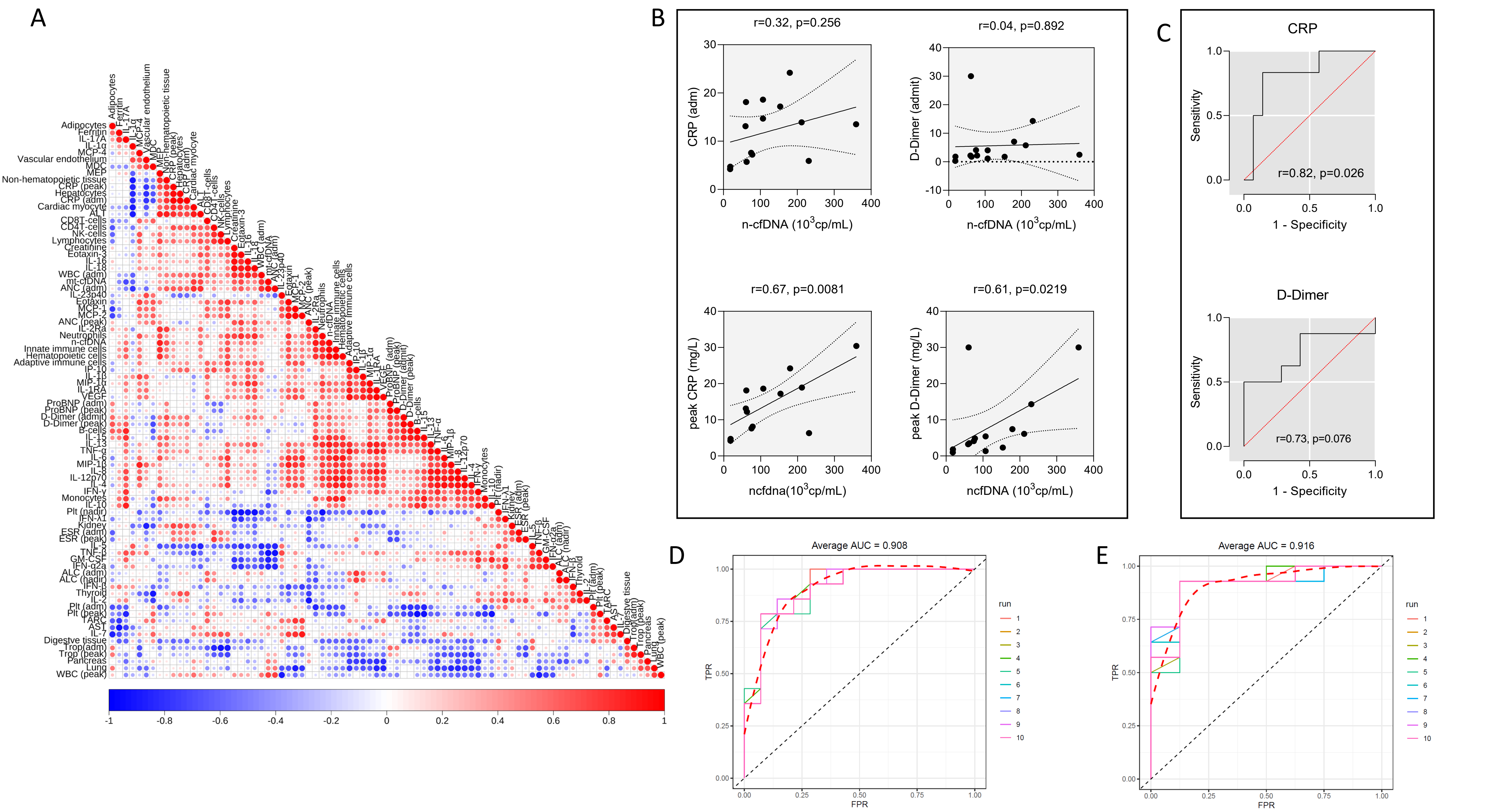
A
Adipocytes
Ferritin
IL-17A
IL-1α
MCP-4
Vascular endothelium
MDC
MEP
Non-hematopoietic tissue
CRP (peak)
Hepatocytes
CRP (adm)
Cardiac myocyte
ALT
CD8T-cells
CD4T-cells
NK-cells
Lymphocytes
Creatinine
Eotaxin-3
IL-16
IL-18
WBC (adm)
mt-cfDNA
ANC (adm)
IL-23p40
Eotaxin
MCP-1
MCP-2
ANC (peak)
IL-2Ra
Neutrophils
n-cfDNA
Innate immune cells
Hematopoietic cells
Adaptive immune cells
IP-10
IL-1β
MIP-1α
IL-1RA
VEGF
ProBNP (adm)
ProBNP (peak)
D-Dimer (admit)
D-Dimer (peak)
B-cells
IL-15
IL-13
TNF-α
IL-6
MIP-1β
IL-8
IL-12p70
IL-4
IFN-γ
Monocytes
IL-10
Plt (nadir)
IFN-λ1
Kidney
ESR (adm)
ESR (peak)
IL-5
TNF-β
GM-CSF
IFN-α2a
ALC (adm)
ALC (nadir)
IFN-β
Thyroid
IL-2
Plt (adm)
Plt (peak)
TARC
AST
IL-7
Digestive tissue
Trop (adm)
Trop (peak)
Pancreas
Lung
WBC (peak)
-1 -0.8 -0.6 -0.4 -0.2 0 0.2 0.4 0.6 0.8 1
B
r=0.32, p=0.256
CRP (adm)
n-cfDNA (10³cp/mL)
r=0.04, p=0.892
D-Dimer (admit)
n-cfDNA (10³cp/mL)
r=0.67, p=0.0081
peak CRP (mg/L)
ncfdna(10³cp/mL)
r=0.61, p=0.0219
peak D-Dimer (mg/L)
ncfDNA (10³cp/mL)
C
CRP
Sensitivity
1 - Specificity
r=0.82, p=0.026
D-Dimer
Sensitivity
1 - Specificity
r=0.73, p=0.076
D
Average AUC = 0.908
TPR
FPR
run
1
2
3
4
5
6
7
8
9
10
E
Average AUC = 0.916
TPR
FPR
run
1
2
3
4
5
6
7
8
9
10

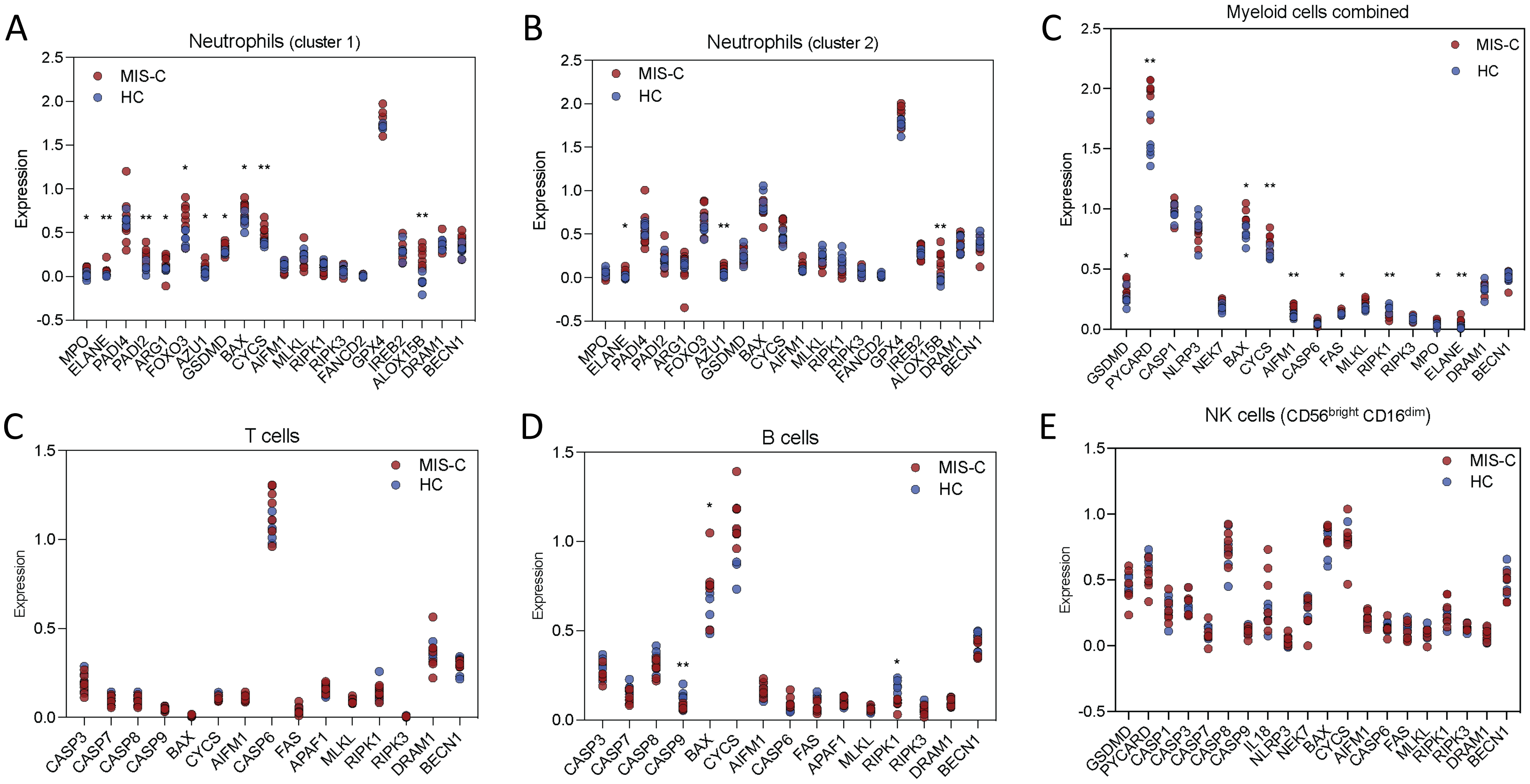
A
Neutrophils (cluster 1)
B
Neutrophils (cluster 2)
C
Myeloid cells combined
C
T cells
D
B cells
E
NK cells (CD56bright CD16dim)
MIS-C
HC
Expression
MPO
ELANE
PADI4
PADI2
ARG1
FOXO3
AZU1
GSDMD
BAX
CYCS
AIFM1
MLKL
RIPK1
RIPK3
FANCD2
GPX4
IREB2
ALOX15B
DRAM1
BECN1
PYCARD
CASP1
NLRP3
NEK7
CASP6
FAS
CASP3
CASP7
CASP8
CASP9
APAF1
IL18

Supplemental Figure Legends

Suppl. Fig 1. Quality of plasma cfDNA and library. (A) Length distribution of plasma cfDNA. (B) Length distribution of cfDNA library.

Suppl. Fig 2. Proportion of cfDNA tissues-of-origin and absolute blood cell count. (A)The proportion of cfDNA derived from hematopoietic cells and non-hematopoietic tissues in MIS-C (red) and pCOVID-19 (blue). (B) Circulating neutrophil and platelet count in MIS-C and pCOVID-19. Mann–Whitney tests utilize to compare groups; ns = not significant.

Suppl. Fig 3. Correlation matrices (within-group) between cfDNA or cytokine features. (A) Correlation between each cfDNA profiles in pediatric patients with and without MIS-C. (B) Correlation between each cytokine profiles in pediatric patients with and without MIS-C

Suppl. Fig 4. Performance cytokine features to differentiate MIS-C from pCOVID-19. ROC curve analysis of cytokine measures early at admission as a discriminatory marker between pCOVID-19 and MIS-C patients.

Suppl. Fig 5. Circulating cytokine levels. Comparison of plasma cytokine/chemokine levels in MIS-C, pCOVID-19 and pHC. Cytokine values are presented as picograms per milliliter (pg/mL; Log_{10} transformed). Statistical significance was determined by kruskal–Wallis test followed by Dunn's multiple comparisons and adjusted for multiple-comparison using the Benjamini-Hochberg procedure. p-value of less than 0.05 was considered statistically significant; *p < 0.05; **p < 0.01; ***p < 0.001; **** p < 0.0001.

Suppl. Fig 6. Association of cfDNA with cytokine and clinical markers. (A) Correlation matrices between cfDNA, cytokine and clinical markers in children with MIS-C. (B) Linear correlation of total n-cfDNA at admission with admission (adm) and peak CRP and D-dimer levels. Correlation coefficients (r) and p-values are shown. (C) Performance by receiver operator curve characteristics of admission CRP and D-dimer to distinguish MIS-C from pCOVID-19. (D) Performance of top five cfDNA measures to distinguish MIS-C from pCOVID-19. (E) Performance of top five features to distinguish MIS-C from pCOVID-19. CRP, D-Dimer and cfDNA features were included in the random forest model. The model was repeated 10 times. The AUC for each repeat or run is shown. Dashed line is the average of the 10 curve.

Figure 7. Increased expression of genes involved in cell death pathways in MIS-C compared to HCs using a publicly available dataset (GSE166489). Single-cell RNA-seq analysis of cell-death transcripts in neutrophil clusters (A & B), myeloid clusters (C), T cells (D), B cells (E) and NK cells (F). Statistical significance was determined by two-tailed t-test after adjusting for multiple comparison using the Benjamini-Hochberg procedure. P-value ≤ 0.05 and FDR≤ 0.25 was considered statistically significant; $^{*}p < 0.05$; $^{**}p < 0.01$; $^{***}p < 0.001$; $^{****} p < 0.0001$.

Suppl. Table 1. Median n-cfDNA in MIS-C patients based on indices of disease severity.

Parameter	On Vasopressor		On Supplemental Oxygen		Intubated	
	Yes (n=7)	No (n=7)	Yes (n=8)	No (n=6)	Yes (n = 2)	No (n=12)
n-cfDNA (cp/mL) median (IQR)	179,790 (107,084 - 231,175)	62,795 (18,528 - 78,249)	166,876 (72,688 - 226,327)	68, 923 (49,443 - 85,458)	210, 340 (61,176 - 359,504)	92, 667 (60,510 - 173,333)

IQR = interquartile range

Suppl. Table 2: High cardiac myocyte-derived cfDNA levels in MIS-C patients compared to pCOVID-19 and healthy controls.

	Cardiac myocyte-derived cfDNA (cp/mL)			
Values	pHC	pCOVID-19	MIS-C	Fold increase
Median [IQR]	152 (105 - 239)	467 (87 - 2361)	3545 (1090 - 9112)	*pHC vs. MIS-C = 23<br>*pHC vs. pCOVID-19 = 3<br>*pCOVID-10 vs. MIS-C = 7.6

Suppl. Table 3: Interpersonal variation of cardiac myocyte-derived cfDNA in MIS-C patients

Subject ID	Cardiac myocyte-derived cfDNA (cp/mL)
SE-JH-H-P0070	3854.1
SE-JH-H-P0068	3235.5
SE-JH-H-P0078	14318.4
SE-JH-H-P0015	10427.8
SE-JH-H-P0042	16942.5
SE-JH-H-P0046	0
SE-JH-H-P0052	8673.8
SE-JH-H-P0004	1114.9
SE-JH-H-P0080	3860.1
SE-JH-H-P0028	2112.2
SE-JH-H-P0044	4481.1
SE-JH-H-P0032	1017.2
SE-JH-H-P0018	565.2
SE-JH-H-P0041	3152.1

Suppl. Table 4: Co-increase of liver-specific enzyme and hepatocyte derived cfDNA in a prototype MIS-C patients

Subject ID	AST u/µL	ALT u/µL	Hepatocyte-derived cfDNA (cp/mL)
SE-JH-H-P0068	585	420	86641
SE-JH-H-P0041	189	338	14635

Reference

47. Reference 48: Ramaswamy A, Brodsky NN, Sumida TS, Comi M, Asashima H, Hoehn KB, et al. Immune dysregulation and autoreactivity correlate with disease severity in SARS-CoV-2-associated multisystem inflammatory syndrome in children. Immunity. 2021;54(5):1083-95.e7.
