# Supplementary material for: Cell-free DNA reveals distinct pathology of multisystem inflammatory syndrome in children
Source: J Clin Invest. 2023 Nov 1;133(21):e171729. doi: 10.1172/JCI171729 (PMC10617770; doi:10.1172/JCI171729)
Supplement: Supplemental Acknowledgments [file jci-133-171729-s216.pdf]

Genomic Research Alliance for Transplantation (GRAfT) investigators, Bethesda, MD

- Sean Agbor-Enoh[1,2,7], MD, PhD
- Pali Shah[2,7], MD, MS
- Jonathan B Orens[2,7], MD
- Palak Shah[3,7], MD, MS
- Shambhu Aryal[4,7], MD
- Steven D Nathan[4], MD
- Michael Eberlein[5,7], MD
- Ananth Charya[5,7], MD, MS
- Michael Keller[1,2,6,7], MD, MS
- Moon Kyoo Jang[1,7], PhD
- Temesgen E Andargie[1,7], PhD
- Hyesik Kong[1], PhD
- Alison F. Davis[7], PHD

Affiliations:

1. Laboratory of Applied Precision Omics, National Heart, Lung, and Blood Institute (NHLBI), NIH, Bethesda, MD
2. Pulmonary and Critical Care Medicine, Johns Hopkins Hospital, Baltimore, Maryland, USA.
3. Inova Heart and Vascular Institute, Inova Fairfax Hospital, Falls Church, VA
4. Advanced Lung Disease and Transplant Program, Inova Fairfax Hospital, Falls Church, VA
5. Division of Pulmonary and Critical Care, University of Maryland School of Medicine, Baltimore, Maryland
6. Department of Critical Care Medicine, National Institute of Health, Bethesda.
7. Genomic Research Alliance for Transplantation (GRAfT), Bethesda, Maryland
